# Physiological assessment of the psychological flow state using wearable devices

**DOI:** 10.1038/s41598-025-95647-x

**Published:** 2025-04-07

**Authors:** Melinda Rácz, Melinda Becske, Tímea Magyaródi, Gergely Kitta, Márton Szuromi, Gergely Márton

**Affiliations:** 1 Institute of Cognitive Neuroscience and Psychology, HUN-REN Research Centre for Natural Sciences, Magyar Tudósok krt. 2, Budapest, 1117 Hungary; 2 School of PhD Studies, Semmelweis University, Üllői út 26, Budapest, 1085 Hungary; 3 Selye János Doctoral College for Advanced Studies, Semmelweis University, Üllői út 22, Budapest, 1085 Hungary; 4 Department of Psychiatry and Psychotherapy, Semmelweis University, Balassa u. 6, Budapest, 1083 Hungary; 5 Mathias Corvinus Collegium, Tas vezér u. 3-7, Budapest, 1113 Hungary

**Keywords:** Psychological flow, Wearable devices, Tetris, Electroencephalography, Photoplethysmography, Motion tracking, Cognitive neuroscience, Psychology, Biomedical engineering

## Abstract

Flow is the state of optimal experience which can lead to outstanding performance. Our study demonstrates the feasibility of detecting and monitoring flow using wearable devices. Twenty-eight Hungarian adults participated in the experiment. They played a game at different levels to induce flow and anti-flow states, which was tested with questionnaires. We measured electroencephalography (EEG), heart rate (HR), blood oxygen saturation (SpO2) and galvanic skin response (GSR) signals as well as head and hand motion. We isolated EEG delta, theta, alpha and beta band power, HR, SpO2 and GSR average and standard deviation, as well as acceleration and angular velocity standard deviation. In flow condition, alpha and theta power were the dominant components, in accordance with the transient hypofrontality hypothesis. We also replicated the U-shaped characteristic of the heart rate variability; in addition, we propose an inverse U-shaped and a U-shaped characteristic for SpO2 and SpO2 variability, respectively. On the basis of motion tracking, subjects were the least physically active in flow, signifying a focused state, and the most active in boredom. Our results support the applicability of lightweight, wearable devices for monitoring mental state that can be utilized to improve well-being at work or in everyday situations.

**Supplementary Information:**

The online version contains supplementary material available at 10.1038/s41598-025-95647-x.

## Introduction

Flow, or optimal experience, means that an individual is completely absorbed in what they are doing^1^; in this experience, the capabilities of the actor are stretched beyond their skills, i.e., actual learning may take place^2^ and outstanding performance can be achieved^3^. Flow is characterized by the following nine dimensions^1^:

Conditions that are inevitable for flow to occur:

1. perceived challenges, or opportunities for action that extend but do not exceed existing skills;
2. clear proximal goals and immediate feedback on the progress made;
3. intense and focused concentration on the present moment;

and accompanying factors when the person is already in the flow state:

4. merging action and awareness;
5. loss of reflective self-consciousness (i.e. loss of self-awareness as a social actor);
6. the feeling that one can control one’s actions; i.e., the feeling that one can, in principle, deal with the situation because one knows how to respond to whatever happens next;
7. distortion of temporal experience (typically, the feeling that time has passed faster than normal);
8. experience of the activity as intrinsically rewarding – an autotelic experience, where the end goal is often just an excuse for the process.

According the first characteristic mentioned above, flow state can occur when the skills of the individual match the requirements of the task. If this condition is not met, the actor may slip into anti-flow states, namely, boredom (if their skills exceed the challenges) or anxiety (if the challenges exceed skills).

## Flow measurement and experimental paradigms

### Measurement methods

Flow measurement has a paradox, namely, that the metacognitive ability of the person experiencing flow is reduced or even absent^4^, their attention is focused on the immediate task and not on themselves and, if they are interrupted, the flow state gets also disrupted^5^. Therefore, flow is most frequently measured after the activity that elicited it.

The Experience Sampling Method is used in studies involving subjects in their natural environment. This mental state assessment tool requires subjects to complete questionnaires at given times (prompted by an electronic device), interrupting their current activity^1,6^. This can be regarded as more practical as information can be collected on subjects embedded in their natural habitat over a longer period of time (studies generally last a few weeks), allowing for the extraction of certain patterns (e.g., behavioural, mood)^6^. Nevertheless, random interruption inevitably leads to disruptions in the flow state, may cause anxiety or embarrassment in subjects, which may hinder the emergence of flow; or may even be dangerous during certain activities requiring caution, such as driving or operating machinery (e.g., in industrial facilities)^6^.

A drawback of questionnaires is that the subjects may not judge their mental state appropriately, or, they may not be able to recall their experiences after the experiment properly. The naturalistic scientific aspect of flow experience by Csíkszentmihályi has become an important focus of recent positive psychological studies^7–10^. Physiological studies are conducted in laboratory conditions to elicit flow and maintain experimental control^11^.

If the research methodology and the factors to be investigated can be clarified, and potential artifacts can be excluded, physiological results can help eliminate the subjective evaluation of the experience. They can contribute to a more precise operationalization of the flow experience, to an “online” exploration of its dynamics, in situ, without interrupting the participant, as the physiological measurement will not affect the fading of self-consciousness as a feature of the flow experience^12–14^. The general methodology of flow measurement is retrospective in nature (interviews, questionnaires), so it would be useful to see the physiological indicators of flow during the experience itself^15^. If the specific patterns that characterize the flow experience are identified, independently of the nature of the task and context used in the study. In this case, the flow experience of the person can be captured without disturbing it, eliminating the methodological problems that arise from the specificity of the phenomenon (fading of self-consciousness, focused attention), as if flow is to be maintained, it is only an afterthought^16^. In recent years, there has been a growing body of research that attempts to assess flow directly, by measuring physiological variables that carry information on the mental state of the subject^13,17–19^.

### Experimental paradigms

Researchers have used a variety of techniques to induce the flow experience, which can be practised in laboratory settings: solving mathematical problems^20^, playing music^21^, computer games^22^, and, particularly, Tetris^23^. Computerized/gamified mental arithmetic^10,13,24–28^ and general cognitive training/assessment tasks such as spatial object tracking^18^, Stroop test^13,29^ and n-back test^30^ are also widely applied. In these activities, the aim has been to induce a relative balance between challenges and skills as a prerequisite of the flow experience^8,22^. By manipulating the level of difficulty of the task^31^, flow and anti-flow states can be compared with their physiological indicators^11,32,33^, then self-report measures can be used to validate these.

The main advantage of games is that the cognitive demand imposed on the subjects can be determined by setting specific parameters (e.g., speed^33^, maps^34^, overall complexity^25,27,30^). A further advantage is the uniformity of the task—although the game may not be the preferred activity of each subject, they are sufficiently engaging and challenging to elicit flow and measurement data can be aggregated and managed uniformly. A third advantage is that these paradigms are only suitable for data acquisition setups for laboratories, e.g., in fMRI-based experiments^19,27^.

One of the most popular games utilized for research purposes is Tetris^23,33,35,36^ and its derivatives^15^, but other genres such as rhythm games^37–39^ and tactical and shooter games^19,40^ have also found their way into flow experiments. We note that the research of dark flow (i.e., flow-like state that leads to maladaptive behaviour patterns) often involves electronic gaming machines^41,42^.

A major drawback of these paradigms is that there is a diversity of flow-inducing activities across individuals; moreover, a more experienced practitioner of a given activity may demonstrate different aspects of flow than a novice^43,44^. Henceforth, in parallel with the automated paradigms, a more personalized direction of research is running, involving specialists in a particular field, such as professional musicians^21,45–47^, dancers^48,49^, athletes^50,51^ or surgeons^52^, not infrequently in the form of case studies^44,46,47,51,53^. These experiments are mostly conducted with the participating professionals at work^47–49^ or practice/rehearsal, sometimes wearing data acquisition devices of interest^21,44–46,48,51^. Some experiments rely on the ‘muscle memory’ of the subjects, using motor imagery tasks^43,53^. Problems with this type of research include the lack of comparable data (i.e., in terms of the paradigm applied and the nature of the activity) and the need for data acquisition devices that are not prohibitive for the subject in question; a potential drawback of using portable devices is that relevant information may escape capture (e.g., the neural activity of the insula, anterior cingulate cortex or basal ganglia cannot be recorded by EEG or fNIRS devices, unlike fMRI).

### Flow and physiological variables

The physiological study of flow is a relatively new field of research. A study by Dietrich^4^ was pioneering as he assumed, frontal lobes may be less active during flow experience, with the regulation of the behavior being rather automatic^54^. This hypothesis has contributed to the development of the concept of effortless attention^55^.

Physiological studies are mostly conducted under laboratory conditions to induce flow and to provide experimental control^11^. Physiological studies of flow have been performed on a number of parameters: brain function, heart rate, respiration, electrodermal response, facial muscle activity, blood glucose, and cortisol levels. Electroencephalogram (EEG) and some brain imaging techniques such as functional magnetic resonance imaging (fMRI)^56^, positron emission tomography (PET)^12,56^ can provide valuable knowledge for psychological studies to understand the functioning of complex higher order brain structures to fully understand psychological phenomenon^57,58^.

### Brain imaging methods

As flow is a psychological construct, the most straightforward way to characterize it is apparently to study the phenomenon in the working human brain. For comprehensive reviews of the neural basis of flow, see^2,54,58,59^. Of the possible neuroimaging methods, functional magnetic resonance imaging (fMRI) provides the broadest view, capable of capturing the activity of all cortical structures. fMRI has been successfully applied in flow research involving mental arithmetic tasks^10,26,27^ and a paradigm applying a first-person shooter game^19^. It also has been the signal acquisition device in a transcranial stimulation experiment^27^. The main advantage of fMRI is that it allows the examination of subcortical structures and certain parts of the cortex deeper under the skull that are involved in the generation of the flow state, such as the putamen^10^, amygdala^10,26,27^, anterior and middle insula^26^, cingulate cortex^19,26^, thalamus^19,26^, caudate nucleus^19,26^, nucleus accumbens^19^, putamen^19^, hippocampus^26^ and the cerebellum^19,26^. The main drawback of this method, beside poor temporal resolution, is that the device requires the subjects to be immobilized, which makes it impossible to study a wide variety of activities that can elicit flow—e.g., playing an instrument, dancing or doing sports.

Functional near-infrared spectroscopy (fNIRS) is a more affordable and portable implementation of blood-oxygen-level-dependent (BOLD) imaging, monitoring blood oxygen levels in surface cortical structures with a lower spatial resolution than fMRI. Although it can be used in research on flow during daily or creative activities^60^, it is still more popular in regular laboratory setups^33,35,36^.

The most widely used portable neuroimaging method is undoubtedly encephalography (EEG), mostly due to its low cost and outstanding temporal resolution. Similarly to fNIRS, EEG can monitor only the activity of superficial cortical areas; making the technique suitable for characterizing the behaviour of the frontal^13,25,50,51,61^, parietal^30,62^, temporal^38,51^ and occipital^62^ cortices affected in generating flow, and the interaction of cerebral structures as reflected in functional connectivity^38,43^. Relevant research also includes testing the hypothesis of transient hypofrontality (i.e., the activity of certain areas of the frontal cortex decreases during flow, corresponding to a decrease in self-awareness^4,63^). Besides being a popular modality in laboratory settings^25,30,38,39,43,50,61^, this technique can also be used to perform flow experiments with physically active subjects^51^. A relatively new branch of research is using portable, easy-to-use EEG headsets such as the Emotiv EPOC^24^ or DSI-24^62^, which is a further step toward non-constraining setups and paradigms that feel natural to the user and do not present an obstacle to flow.

### Cardiovascular characterization

The mechanisms involved in generating flow are not limited to the brain: the sympathetic and parasympathetic branches of the autonomic/visceral nervous system also contribute to the state and this is reflected in certain physiological variables, most notably heart rate (HR) and heart rate variability (HRV). Studies show that HR positively correlates with arousal^36,37^ and a moderate HR is required for flow^15^. HRV is negatively correlated with flow; to be more specific, there is a U-shaped characteristic between flow and HRV (i.e., HRV is greater during boredom and stress than in flow)^36^. Most importantly, the high-frequency (HF, 0.15–0.4 Hz) component of HRV, which reflects vagal tone (parasympathetic activity)^64–66^, is reduced during flow^18,29,33,45^; it has also been shown that subjects with shyness (along with a heightened predisposition for introspection and anxiety), tend to experience lower levels of flow^15^. A similar quadratic dependence of flow on the low-frequency (LF, 0.04–0.15 Hz) component of HRV, corresponding to sympathetic^64^ or both sympathetic and parasympathetic^65,66^ activity, has also been demonstrated^29^. As for the LF/HF ratio, a measure of sympathovagal balance^64–66^, mixed results are reported^21,29^.

Besides the variables mentioned above, other cardiovascular measurements have been successfully applied in flow research, such as blood volume pulse^13,24^, interbeat interval^24^, blood pressure^21,23^, impedance cardiography^23^ and thoracocardiography^48^ signals.

These variables are most commonly measured by electrocardiography (ECG)^15,18,23,34,39,41,51^ or photoplethysmography (PPG). In recent years, portable/wearable solutions have been introduced for both modalities such as the Biosignalplux RespiBAN^24^, Polar V800^45^, H7^29^ and H10^37^, BIOPAC BioNomadix^33^ and BioHarness^36^, VivoMetrics LifeShirt^48^, Shimmer Sensors^44^ (ECG), Empatica E4 wristband^24^ and Fitbit Versa 3^62^ (PPG).

### Skin conductance and other modalities

Besides cardiovascular variables, electrodermal activity (EDA)–also referred to as galvanic skin response (GSR) or, simply, skin conductance (SC) in the literature—is also a feasible and widely used indicator of mental states. Skin conductance is positively correlated with arousal^13^ and its moderate level has been reported to co-occur with flow state^15^: in this case, an inverse U-shape characteristic is suggested^26^.

Besides traditional laboratory data acquisition devices^13,26,34,67^, portable/wearable solutions have gained importance in monitoring electrodermal activity; such devices include BIOPAC BioNomadix^33^ and Shimmer Sensors^44^.

Other biological variables relevant to flow research but not discussed in detail here, include respiratory metrics such as respiratory period and depth^13,15,21,24,29,33,34,39,48^, skin temperature^24^, cortisol level^40^, electromyographic signals^13,21,24,51^, accelerometric signals^21,48^ and pupil size^13,30^.

The aim of the present study is to explore the physiological parameters of flow during Tetris, with optimal settings of difficulty levels—to be able to reproduce flow and anti-flow (e.g., boredom and anxiety) states. In Tetris, participants need to adjust the orientation of pieces of 2D blocks in different shapes to complete full lines at the bottom of the playing screen^23^. Our aim is to replicate previous research—see Table 1—on a Hungarian sample^24,34,69,70^.

**Table 1:** Previous physiological flow studies with the game Tetris.

Study	Measure	Results
^68^	EDA, BP, Res, T	Increase in EDA & HR with increasing difficulty
^69^	HRV, Cortisol	Lower HRV & higher cortisol in the flow condition
^33^	fNIRS, ECG, Res	Larger RD & lower LF-HRV in the flow condition
^23^	ICG, ECG, BP	Weak relationship between self-reported flow and CV indices of relative challenge, relative challenge correlated positively to flow
^14^	HRV, ECG, cortisol	Inverted U-shaped relationship of flow with the values of sympathetic arousal and cortisol. Moderate sympathetic arousal and HPA-axis activation can be related to flow during a tasksolving
^15^	Res, HR, HRV, SC	Flow and faster respiratory rate, deeper Res, moderate HR, moderate HR variability, and moderate SC

EDA: electrodermal activity; BP: blood pressure; T: temperature; HR: heart rate; HRV: heart rate variability; LF-HRW: low-frequency heart-rate variability; RD: respiratory depth; fNIRS: functional near-infrared spectroscopy; ECG: electrocardiography; Res: respiration; ICG: impedance cardiographic signals; SC: skin conductance.

A previous study found a U-shaped relationship between flow and psychological arousal—boredom corresponds to moderate arousal and stress (anxiety as an anti-flow experience) to higher levels of arousal, while flow corresponds to moderate level of psychological arousal^70^. During a task-solving situation, the flow experience represents moderate mental effort due to increasing parasympathetic modulation of sympathetic activity^15^.

### Hypotheses

In general, we hypothesize that there are significant differences between flow and anti-flow (anxiety, boredom)^71^ situations in terms of physiological factors of participants that can be assessed using wearable signal acquisition devices with parameters theoretically inferior to their counterparts in laboratory settings. In addition, we propose the following hypotheses based on prior literature:

- H1: brain activity is reduced in flow (theta and alpha enhancement^25,63^, beta power reduction^38,50^);
- H2: average HR is lowest in boredom and highest in frustration^15,36,37^;
- H3: HRV is lowest in flow^36^;
- H4: GSR is highest in boredom and lowest in frustration^13,15,16^;
- H5: there is difference between motion states of subjects in experimental conditions (despite the findings of^21^).

## Results

### EEG bandpower

**Fig. 1:**
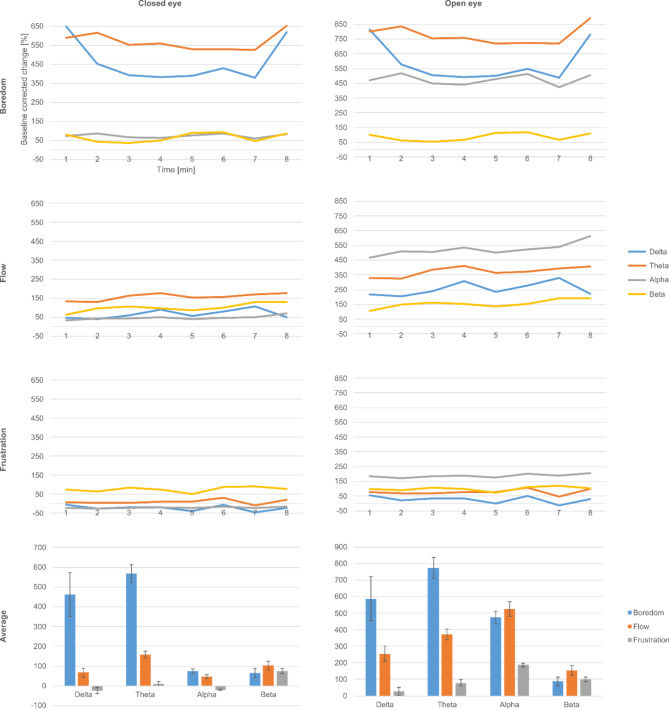
Changes in EEG power during the experiment. First three rows: time dependence of the power change in EEG frequency bands relative to eyes-closed (left column) and eyes-open (right column) baseline under the experimental conditions. Bottom row: Average power change measured in the frequency bands under the experimental conditions.

Average EEG power showed distinctly different behavior in the three experimental situations over the time course of the measurements. Upon the application of eyes-closed baseline correction, the following results were obtained. In boredom, theta and—to a lesser extent—delta power increased remarkably, exhibiting approximately six- and fivefold increases, respectively. Alpha and beta power nearly doubled in this condition. In flow, a significantly smaller power increase was observed in the theta and delta bands, and a slightly smaller increase in the alpha band. Beta band power, however, slightly increased compared to boredom. In frustration, delta and alpha power decreased compared to baseline and theta exhibited only a negligible increase. Beta power nearly doubled, similarly to the other conditions. The left column of Fig. 1 shows power changes in each frequency band under the experimental conditions. We found significant differences between boredom and flow in the alpha and beta bands, between flow and frustration in the delta, theta and alpha bands, and between boredom and frustration in the delta band. For details, see Table 2.

**Table 2:** Significant differences in closed-eye baseline-corrected EEG between conditions, for each channel and frequency band.

	Statistic (t)	df	CI_95%, low_	CI_95%, high_	Estimate (mean)	*p*-value
Boredom-flow, AF7, alpha	− 2.7408	15	− 18.3633	− 2.2968	− 10.33	0.0152
Boredom-flow, AF7, beta	− 3.5495	15	− 21.7564	− 5.4309	− 13.5936	0.0029
Boredom-flow, Fp1, beta	− 2.3785	15	− 16.7516	− 0.9175	− 8.8346	0.0311
Boredom-flow, Fp2, beta	− 2.2918	14	− 18.8996	− 0.6261	− 9.7629	0.0379
Flow-frustration, Fp1, theta	2.2049	16	0.3144	16.0014	8.1579	0.0424
Flow-frustration, Fp1, alpha	2.1717	16	0.1904	15.7873	7.9889	0.0453
Flow-frustration, AF8, delta	2.5743	15	1.7468	18.5614	10.1541	0.0212
Flow-frustration, AF8, theta	2.5912	15	2.2231	22.8354	12.5293	0.0205
Flow-frustration, AF8, alpha	2.4969	15	1.7113	21.6708	11.6911	0.0247
Boredom-frustration, AF7, delta	2.4723	17	1.265	15.9913	8.6281	0.0243

For eyes-open baseline correction, we obtained the following results. Boredom delta, theta and beta increased at approximately the same rate as in the eyes-closed condition, only to a greater extent. In contrast, alpha power increased fivefold. Alpha power also increased to the same extent in the flow condition, becoming the dominant frequency band in this state, followed by theta power. Alpha power was also the most prominent component in frustration, but its increase was considerably smaller. Interestingly, beta power nearly doubled in each condition and this effect was the strongest in flow. For more details, see the right column of Fig. 1. We found significant differences between both flow and frustration, and boredom and frustration in the delta, theta and alpha bands; we found no significant difference between boredom and flow in this case. For details, see Table 3.

**Table 3:** Significant differences in open-eye baseline-corrected EEG between conditions, for each channel and frequency band.

	Statistic (t)	df	CI_95%, low_	CI_95%, high_	Estimate (mean)	*p*-value
Flow-frustration, Fp2, delta	2.7498	15	2.7165	21.4426	12.0795	0.0149
Flow-frustration, AF8, delta	3.342	15	5.318	24.0447	14.6814	0.0045
Flow-frustration, AF8, theta	2.4629	15	1.8609	25.7951	13.828	0.0264
Flow-frustration, AF8, alpha	2.4402	15	1.699	25.159	13.429	0.0276
Boredom-frustration, AF7, delta	2.3191	16	0.8091	18.0281	9.4186	0.0339
Boredom-frustration, AF8, delta	3.3052	12	5.1254	24.9533	15.0393	0.0063
Boredom-frustration, AF8, theta	2.9022	12	4.2432	29.8019	17.0225	0.0133
Boredom-frustration, AF8, alpha	2.5397	12	1.9797	25.883	13.9313	0.026

### PPG variables

**Fig. 2:**
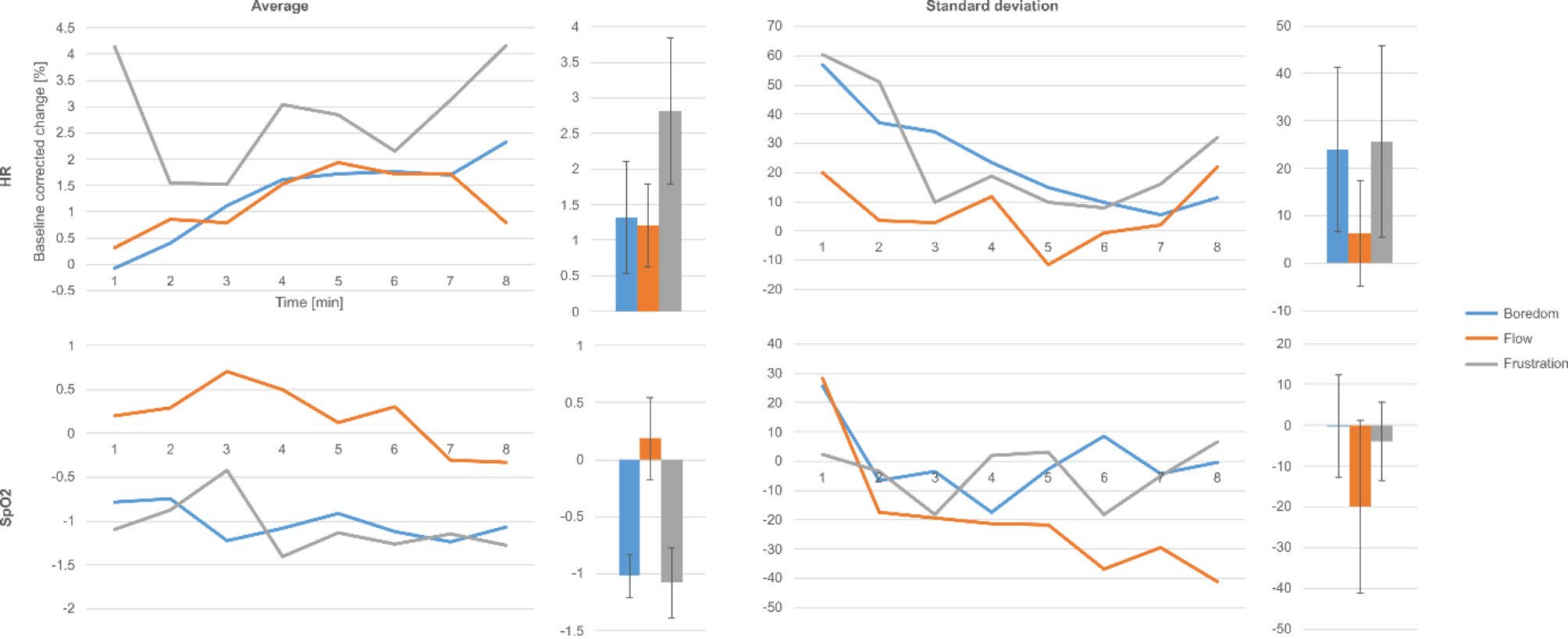
Time dependence of PPG variables. Values are relative to eyes-closed baseline across the experimental situations. The top row refers to heart rate, the bottom to blood oxygen saturation; the left column shows averages, the right standard deviations. The average and standard deviations of the curves are shown on the right of each graph.

Average HR across the subjects showed the greatest changes in frustration, followed by boredom and flow, with these latter conditions being nearly indistinguishable in HR magnitude. The shape of the curves, however, showed some differences, with boredom HR increasing monotonously and flow HR increasing until minute 5, then decreasing. Average HR is shown in the top left subfigure of Fig. 2. Average SD, roughly corresponding to HRV, showed a U-shaped characteristic over time under flow and frustration conditions, being the highest at the beginning of the trials, gradually decreasing, then slightly increasing toward the end of the measurement. Boredom SD, however, decreased linearly during almost the entire course of the trial, showing a small increase only in the last minute. Nevertheless, the range of boredom SD largely overlapped with that of frustration, while flow SD was considerably smaller. For details, see the top right subfigure of Fig. 2. Significant differences between all three conditions were found in HR average. HR SD showed significant differences in flow and frustration. For details, see Table 4.

**Table 4:** Significant differences in heart rate average (AVG) and standard deviation (SD) between conditions.

	Statistic (t)	df	CI_95%, low_	CI_95%, high_	Estimate (mean)	*p*-value
Boredom-flow, AVG	2.6464	18	0.349	3.0373	1.6931	0.0164
Flow-frustration, AVG	− 2.3323	17	− 3.666	− 0.1836	− 1.9248	0.0322
Flow-frustration, SD	− 4.9285	17	− 38.6103	− 15.4626	− 27.0365	0.0001
Boredom-frustration, AVG	− 2.3463	19	− 4.5888	− 0.2618	− 2.4253	0.03

SpO2 average only showed small positive changes in flow. Boredom and frustration SpO2 exhibited nearly identical characteristics, along with a 1% decrease from baseline. SpO2 variability exhibited small values around zero for boredom and frustration and a strongly decreasing trend in flow. The characteristics for SpO2 average and standard deviation are shown in the bottom left and right subfigures of Fig. 2, respectively. Differences between conditions did not reach the level of significance.

### Galvanic skin response

**Fig. 3:**
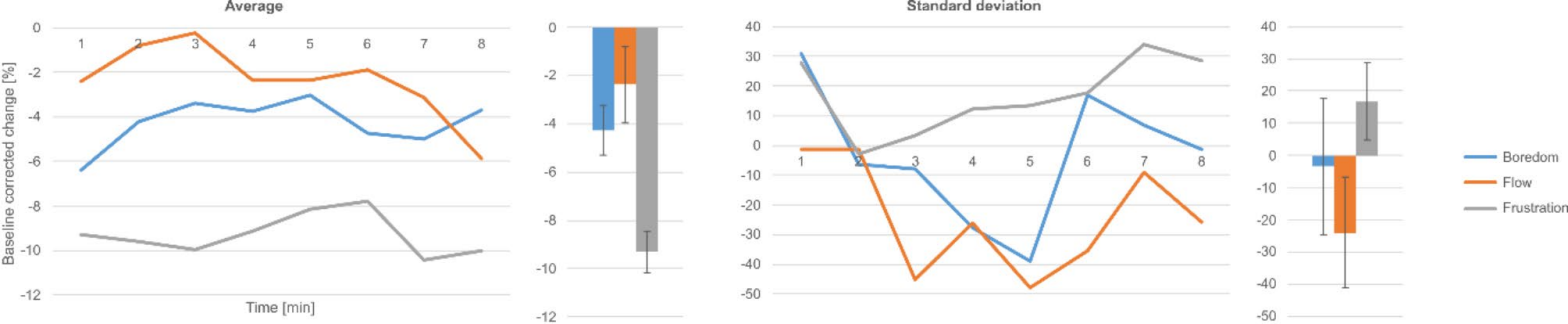
Changes in GSR variables during the experiment. Time dependence of the average (left) and the standard deviation (right) of the GSR relative to the closed eye baseline in the experimental situations.

Average skin impedance decreased from baseline in all experimental situations, with flow exhibiting the lowest and frustration the largest changes. GSR variability proved to be the lowest in flow and highest in frustration. GSR mean and standard deviation are shown in Fig. 3. Differences between conditions did not reach the level of significance.

### Physical activity

**Fig. 4:**
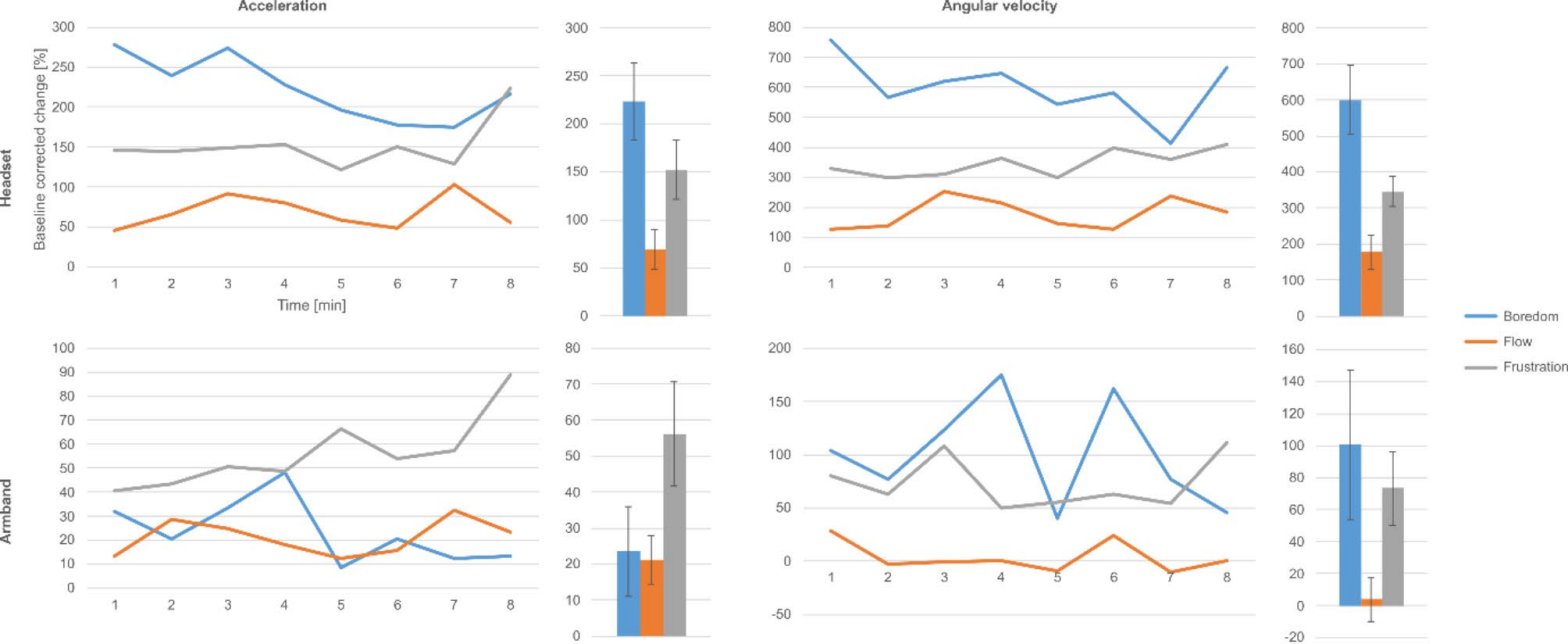
Changes in physical activity during the experiment. Time dependence of the standard deviation of IMU variables relative to eyes-closed baseline across the experimental situations. The top row refers to the headset, the bottom to the armband; the left column accelerometer data, the right angular velocity.

The lowest degree of physical activity was observed in flow, and, interestingly, highest in boredom (except for armband acceleration, where frustration was dominant). Average acceleration and angular velocity SD measured by the two devices are shown in Fig. 4. Significant differences were observed regarding both devices between boredom and flow (except for acceleration measured by the armband), and frustration and flow. For details, see Table 5.

**Table 5:** Significant differences in acceleration (ACC) and angular velocity (AV) between conditions for the headset (HS) and the armband (AB).

	Statistic (t)	df	CI_95%, low_	CI_95%, high_	Estimate (mean)	*p*-value
Boredom-flow, ACC, HS	3.3637	21	125.7712	533.1406	329.4559	0.0029
Flow-frustration, ACC, HS	− 2.5375	21	− 259.1715	− 25.7005	− 142.436	0.0192
Flow-frustration, ACC, AB	− 4.2572	21	− 165.6444	− 56.922	− 111.2832	0.0004
Boredom-flow, AV, HS	4.3119	21	218.4472	625.4613	421.9543	0.0003
Boredom-flow, AV, AB	2.1094	19	0.3718	95.3546	47.8632	0.0484
Flow-frustration, AV, HS	− 3.7411	21	− 352.0085	− 100.4794	− 226.244	0.0012
Flow-frustration, AV, AB	− 5.0703	19	− 85.5473	− 35.5557	− 60.5515	0.0001

## Discussion

In this study, we attempted to assess the physiological correlates of flow state using wearable devices, namely, EEG power in the delta, theta, alpha and beta frequency bands above the prefrontal cortex, PPG variables such as heart rate and blood oxygen level, skin impedance and variables of motion state such as acceleration and angular velocity.

According to the transient hypofrontality hypothesis, brain activity shows a decrease in flow; more specifically, power in the theta and alpha bands increases^25,63^ and beta power decreases^38,50^. In accordance with the previous statement, we were able to demonstrate the dominance of the alpha and theta power in flow. Nevertheless, we also observed that delta and alpha power also increases in boredom condition (in fact, theta increase is actually stronger in boredom than in flow and alpha increase is nearly the same in the two conditions); this may be due to the fact that the majority of the participants did not play Tetris regularly and some of them may have also experienced flow in the game variant designed to elicit boredom as^23^ a hypothesis. In addition, some players attempted to make the game more stimulating by playing less efficiently, e.g., stacking tetrominoes instead of filling the rows at the bottom of the field. It is worth noting that this only applies to the open eye baseline corrected data; a possible explanation for this is that subjects generate more frontal theta and alpha power during eyes-closed rest than during eyes-open rest. A more surprising finding was that beta power was the most prominent in flow compared to the other two conditions, regardless of baseline type.

Prior literature has shown that HR positively correlates with arousal^36,37^, resulting in moderate flow HR^15^. In our experience, boredom and flow HR proved to be almost identical, with flow HR being slightly lower. This seems to support the hypothesis that in boredom condition, the majority of subjects experienced a state similar to flow. Consistent with a stronger arousal, frustration HR proved to be the substantially larger than either flow or boredom HR. Consistent with the findings of^36^, our results indicate that HRV (or, more specifically, HR standard deviation) reached its minimum during flow; moreover, HRV magnitude was nearly the same in boredom and frustration, resulting in a U-shaped curve (see the top right subfigure of Fig. 2).

As PPG is mentioned in prior literature only as a non-invasive way to measure ECG, we did not formulate a hypothesis on blood oxygen saturation. Nevertheless, we found that similarly to HRV, flow can be distinguished from boredom and frustration using both SpO2 average and standard deviation. Flow average SpO2 increased weakly with decreasing variability, while boredom and frustration SpO2 showed a slight reduction, with their variability varying around zero, yielding an inverted U-shaped characteristic for SpO2 and a U-shaped characteristic for SpO2 variability (with arousal as the independent variable, see the bottom left and the bottom right subfigures of Fig. 2).

Previous works^13,15,16^ have shown that GSR is negatively correlated with arousal. We partially failed to reproduce these results: we found frustration GSR to be the lowest; however, the highest values were measured in the flow condition instead of boredom. Moreover, flow values were the most consistent, exhibiting the least variability.

There are a limited body of studies that use motion tracking in flow analysis. De Manzano et al.^21^ found no significant difference between flow and non-flow states when analyzing the head movements of pianists. In our case, all subjects used two devices equipped with IMUs (i.e., the headset and the armband) and were instructed not to move too much in order to avoid electromyographic or contact artifacts. We found that this was the easiest to follow in flow; probably due to the focused presence of the subject, this condition recorded the least amount of movement in both devices. Interestingly, subjects were the most active in the boredom condition in general; a possible explanation for this is that they—either consciously or unconsciously—tried to maintain a higher level of arousal.

The study faced the following limitations. The neural correlates of flow are most commonly assessed using EEG devices intended for laboratory use, i.e., they are capable of acquiring data on multiple channels and their electrodes have much lower contact impedance. As we utilized a lightweight headband with only four channels, we were only able to characterize the behaviour of a smaller part of the cortex. Signal quality became also a crucial factor, as we applied stainless steel electrodes with electrical properties inferior to platinum/iridium^72^ and Ag/AgCl^73–75^ used in other EEG headbands^76,77^. Another limitation is the small sample size, which was also compromised by data loss and corruption due to contact issues. Our sample cannot be regarded as representative, as we applied convenience sampling, resulting in the majority of subjects being Hungarian-speaking undergraduates.

Studying flow from a physiological focus and revealing the latent pattern behind this optimal experience can help professionals identify this state and facilitate it through biofeedback or use the results in any other interventions or developmental procedures. We aimed to contribute to this aim with this pilot study.

## Methods

### Sample

Twenty-eight Hungarian healthy adult subjects participated in the study (we used a convenience sampling method). Participation was anonymous and voluntary. The mean age was 27.60 (*SD* = 8.23). The demographic characteristics of the sample are shown in Table 6.

**Table 6:** Demographic characteristics (and practice level of Tetris) (*N* = 28).

Variable	%
Gender	
Male	53.6
Female	46.4
Education	
College student	21.4
College graduate	67.9
Postgraduate	10.7
Residency	
Capital city (Budapest)	82.1
City	7.1
Town, village	7.1
Abroad	3.6
Relationship status	
Single	42.9
In a relationship	57.1
Practice in Tetris	
Tried 1–2 times	78.6
Regularly	21.4

### Measures

#### Instruments

For signal recording, we used two wearable devices developed by the Mathias Corvinus Collegium—an EEG headset and an EMG armband integrating PPG functionality.

The headset is capable of capturing EEG signals over four channels over the prefrontal/frontal cortex, with locations corresponding to standard (i.e., according to the 10–20 system) positions AF7, Fp1, Fp2 and AF8. Reference and driven right leg electrodes are placed behind the left and right ear, respectively. EEG signals are sampled at 250 Hz and 24-bit (0.045 µV) resolution. The device also includes an inertial measurement unit (IMU) to allow motion tracking. The IMU contains a 3-DOF accelerometer and gyroscope operating at 50 Hz and 16-bit (0.061035 · 10^− 3^ g and 0.01526 dps, respectively) resolution.

The EMG and IMU modules of the armband are identical to the EEG and IMU units of the headset, except that the EMG module can record signals on six channels; this feature was not used in our research. The device also implements GSR measurement at 500 Hz and 12-bit resolution (0.61 ohm). The integrated PPG unit provides HR and SpO2 level information at 8-bit resolution (1 BPM and 1%, respectively).

**Fig. 5:**
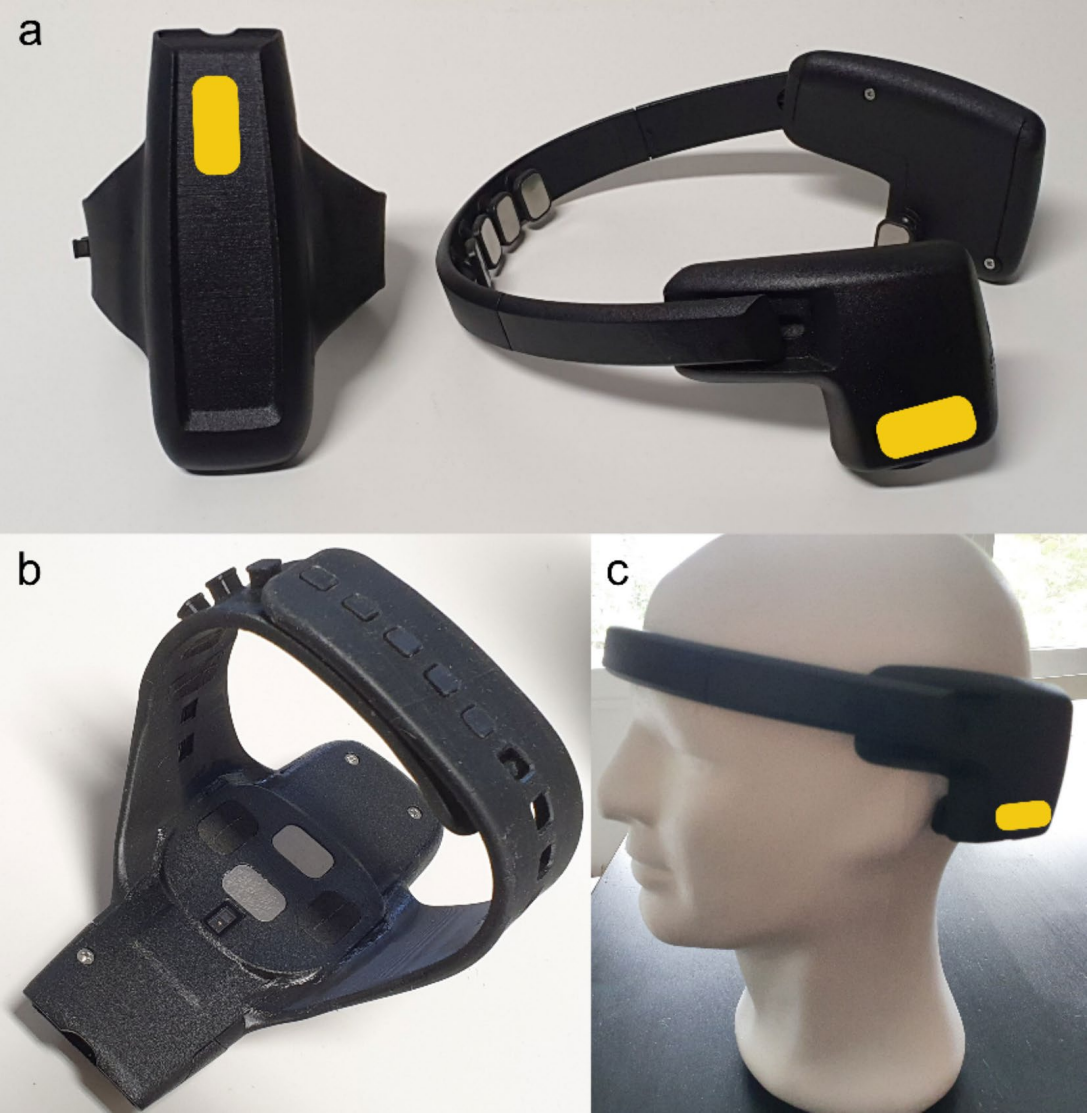
Photographs of the devices used in this study. (**a**) Top view of the armband (left) and side view of the EEG headset (right). (**b**) Bottom view of the armband with the PPG sensor and the steel electrodes of the galvanic skin response measurement circuit visible; c) view of the intended use of the headset.

The devices used in the research are shown in Fig. 5.

The devices were connected to a Raspberry Pi 3 via UDP; this Pi merged and channeled incoming data to a host computer where actual data acquisition took place. Data management scripts running on the Pi and the host PC were implemented in Python.

EEG of the participants was recorded using the headset; GSR, HR and SpO2 using the armband. However, the armband did not provide adequate GSR data for the forearm; therefore, it was not utilized thereon, but instead used as follows. The device was placed on the table in front of the subject and the left index finger was placed on the GSR and PPG sensors. An elastic band was attached to the device to avoid accidental removal of the finger. In addition, accelerometers and gyroscopes of the two devices were used to assess the physical activity of the subjects across the experimental situations. Screenshots were also taken on the host PC running the experimental paradigm with the informed consent of the subjects using the standard Snipping Tool for Windows. These recordings were not further analyzed.

#### Experimental task

Tetris is a video game in which the user controls the movement of different shapes consisting of four squares (tetrominoes) that fall from the top of the playfield to the bottom (or, if it is not empty, on the top of other tetrominoes); the user can move and rotate them horizontally, sending them down to the bottom, and may speed up the game with the keyboard keys. When a row of squares has been completed, it disappears; when the pile of tetrominoes reaches the top of the field, the game is over. The speed can also increase as the game progresses: when the number of rows removed reaches 20 times the actual level, the level increases (saturating at 9), resulting in a proportional speed adjustment.

In this research, the Tetris implementation also used in^33^ was applied. We modified the basic version (available at https://www.percederberg.net/games/tetris/index.html) written in Java as follows:

- in the version inducing boredom, the speed was set to a lower, fixed level (1 s/square, so that a tetromino fall takes 20 s assuming an empty field); players could not drop tetrominoes to the bottom;
- in the version corresponding to flow state, we left the game untouched, giving the participants full control over the game. The initial speed of the game was 0.5 s/square;
- in the version eliciting frustration, the initial speed was set to a faster level (0.15 s/square), which could be further increased. The direction of rotation was set to random.

### Questionnaires

This laboratory study focused on the psychological biological aspects of optimal experience. In addition to recording physiological markers, participants in each experimental phase completed different questionnaires on related mood indicators and their flow and anti-flow state characteristics.

#### Shortened positive and negative affect schedule (PANAS)

The Hungarian version of the inventory^78^ was developed based on Positive and Negative Affect Schedule of Watson et al.^79^. It has two dimensions: five items each for positive and negative affectivity, with items on a five-point Likert scale (from 1: Not at all to 5: Very much). The reliability indexes of the scales are acceptable (positive affect: α = 0.73, negative affect: α = 0.62).

#### Flow state questionnaire of the positive psychology lab (PPL-FSQ)

PPL-FSQ^80^ measures flow experience at a state level for a specific activity across 20 items. Subjects rate the items on a 5-point Likert scale (from 1: Strongly disagree to 5: Strongly agree). It has two dimensions: the balance between challenges and skills scale with 11 items (basic conditions of flow) and the absorption in the activity factor with 9 items (the accompanying characteristics of flow). The internal consistency of the scales is acceptable (balance between challenges and skills: α = 0.92; absorption in the task: α = 0.91).

### Procedure

The participants received both verbal and written explanations of the pilot protocol. Informed consent was obtained from all participants. All experimental protocols were approved by the United Ethical Review Committee for Research in Psychology (Approval No. 2023 − 106). All methods were performed in accordance with the Declaration of Helsinki, and with relevant guidelines and regulations.

The experiment was carried out in closed, laboratory conditions. A repeated measures design was used. We avoided sequence effects by randomly changing the order of the experimental conditions.

After participants signed the informed consent form, we installed the equipment, and started screen recording to monitor the performance of the subjects during the game. The study started with a questionnaire (questions on demographics and practice in Tetris, baseline mood, performance motivation and dispositional flow), followed by a 2-minute baseline registration: 1 min with eyes open and 1 min with eyes closed. Participants had the opportunity to practice Tetris for 2 min.

**Fig. 6:**
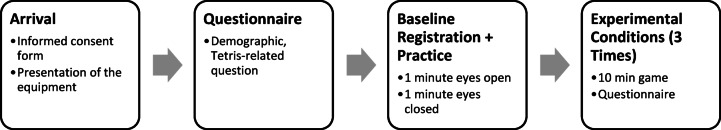
The procedure of the experiment.

The baseline registration was followed by an experimental setting of Tetris, in random order (flow means optimal; frustration means too difficult as speed is high; boredom means too easy, the participant cannot regulate the speed, which is slow). In each setting, participants played Tetris for 10 min and then answered questions on their experiences (see Fig. 6). Altogether, the duration of the whole experiment was 45 min per person.

### Data preprocessing

Physiological data were processed with scripts written in Python 3.9, utilizing libraries NumPy (version 1.25.1), SciPy (version 1.11.2) and scikit-learn (version 1.4.0).

Data from four subjects were excluded from the analysis due to problems encountered during recording. Data properly recorded were partitioned into five phases corresponding to open and eyes-closed baselines and experimental situations. Manual examination of the phase lengths for further analysis resulted in 60 (1 min, baseline) and 480 (8 min, experimental situation) seconds. These phases were positioned within the data so that the shortest baseline/experimental data block for all the subjects would be preceded and succeeded by the same amount of unused data.

### EEG data

EEG data were filtered between 0.5 and 30 Hz using second order Butterworth filters. Bad channels were selected by manual inspection and removed from further analysis. Then blinking and other artifacts were eliminated from the data using independent component analysis. Subsequently, experimental data were partitioned into 1-minute segments and the Fourier transform of these segments was taken (along with the baseline). Parts of the power spectrum pertaining to frequency bands (delta: 0.5–4 Hz, theta: 4–7 Hz, alpha: 8–12 Hz, beta: 13–30 Hz) were aggregated. Next, the average of the data of all participants was calculated (with outliers removed before aggregation).

### PPG data

Contact artifacts were eliminated from HR and SpO2 data as follows. SpO2 data outside the [70%, 100%] range specified for valid measurements by the PPG sensor datasheet were replaced with the average of the valid values of their left- and right-hand sides. The range/threshold of HR artifacts was characterized separately for each subject upon manual examination; incorrect values were replaced similarly to their SpO2 counterparts. Then, experimental data were partitioned into 1-minute segments and the mean and standard deviation (SD, we assessed HRV with the standard deviation of HR) of these segments were calculated, together with the baseline. Finally, the average of these values across all subjects were also calculated. Two subjects yielded incomplete data in some experimental situations (one each during flow and frustration), so correction was not possible; these data were excluded from data aggregation. Outliers were also excluded from analysis.

### GSR data

Experimental data were partitioned into 1-minute segments and the mean and SD of these segments were calculated, together with the baseline. Then, the average of the values for all subjects were also calculated. Five subjects yielded incomplete data in some experimental situations (five during flow and four during frustration), so correction was not possible; these data were excluded from data aggregation. Outliers were also excluded from analysis.

### IMU data

Experimental data from the EEG headset and the armband were partitioned into 1-minute segments. The magnitude of the instantaneous acceleration and angular velocity were calculated by taking their Euclidean norm. Then, the SD of the experimental segments and baseline data were calculated. Finally, the average of these values for all subjects were also calculated. Outliers were excluded from analysis.

### Baseline correction

All data presented in the following section underwent baseline correction, which involved data recorded with the eyes open or closed of the subjects. We applied Eq. (1) for correction.

1 $$\:{x}_{BC}=\frac{x-{x}_{BL}}{{x}_{BL}}\cdot\:100\%,$$

where $\:{x}_{BC}$ is the baseline corrected value, $\:x$ is the raw value and $\:{x}_{BL}$ is the eyes-open or eyes-closed baseline data.

As the type of baseline data may affect results, we applied both eyes-open and eyes-closed baseline correction for EEG data. Contrary to this, as eyes-closed baseline corresponds to the most relaxed state possible, we used only eyes-closed baseline correction on PPG, GSR and IMU data.

### Statistical analysis

The difference between experimental conditions was assessed using one-sample t-tests. Significance level was set to 0.05. Statistical analysis on EEG data was performed on the log-transform of each channel separately. All scripts were written in R^81^. Outliers were detected and removed using QQ plots implemented in the Companion to Applied Regression (car)^82^ package prior to the analysis.

## Electronic supplementary material

Below is the link to the electronic supplementary material.

Supplementary Material 1

## Data Availability

Code, additional figures and tables are provided as supplementary material to this article. The accompanying dataset is available at https://hdl.handle.net/21.15109/ARP/968SUZ.
