# Supplementary figures and images for: Physiological assessment of the psychological flow state using wearable devices (part 2 of 2)
Source: Sci Rep. 2025 Apr 7;15:11839. doi: 10.1038/s41598-025-95647-x (PMC11977251; doi:10.1038/s41598-025-95647-x)

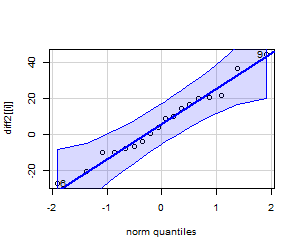

Supplement: Supplementary file 1 — Supplementary Material 1 [file 41598_2025_95647_MOESM1_ESM.zip › Supplementary/QQplot_26ch3_closed_theta_theta_flow - ch3_closed_theta_theta_frustration.png]

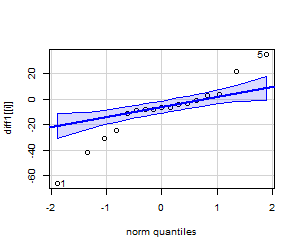

Supplement: Supplementary file 1 — Supplementary Material 1 [file 41598_2025_95647_MOESM1_ESM.zip › Supplementary/QQplot_27ch3_closed_alpha_alpha_boredom - ch3_closed_alpha_alpha_flow.png]

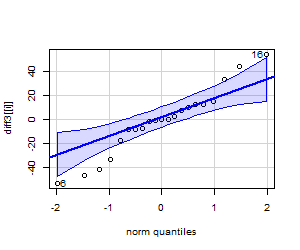

Supplement: Supplementary file 1 — Supplementary Material 1 [file 41598_2025_95647_MOESM1_ESM.zip › Supplementary/QQplot_27ch3_closed_alpha_alpha_boredom - ch3_closed_alpha_alpha_frustration.png]

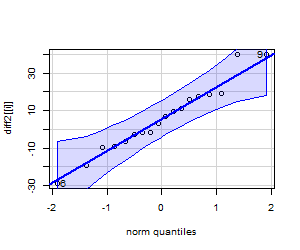

Supplement: Supplementary file 1 — Supplementary Material 1 [file 41598_2025_95647_MOESM1_ESM.zip › Supplementary/QQplot_27ch3_closed_alpha_alpha_flow - ch3_closed_alpha_alpha_frustration.png]

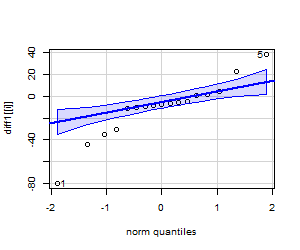

Supplement: Supplementary file 1 — Supplementary Material 1 [file 41598_2025_95647_MOESM1_ESM.zip › Supplementary/QQplot_28ch3_closed_beta_beta_boredom - ch3_closed_beta_beta_flow.png]

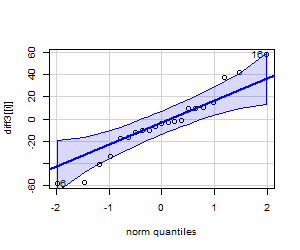

Supplement: Supplementary file 1 — Supplementary Material 1 [file 41598_2025_95647_MOESM1_ESM.zip › Supplementary/QQplot_28ch3_closed_beta_beta_boredom - ch3_closed_beta_beta_frustration.png]

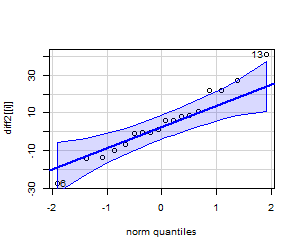

Supplement: Supplementary file 1 — Supplementary Material 1 [file 41598_2025_95647_MOESM1_ESM.zip › Supplementary/QQplot_28ch3_closed_beta_beta_flow - ch3_closed_beta_beta_frustration.png]

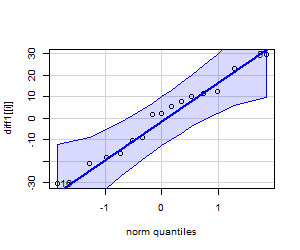

Supplement: Supplementary file 1 — Supplementary Material 1 [file 41598_2025_95647_MOESM1_ESM.zip › Supplementary/QQplot_29ch4_closed_delta_delta_boredom - ch4_closed_delta_delta_flow.png]

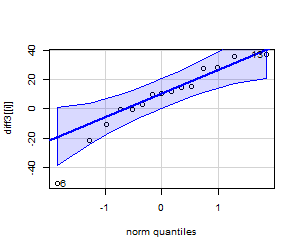

Supplement: Supplementary file 1 — Supplementary Material 1 [file 41598_2025_95647_MOESM1_ESM.zip › Supplementary/QQplot_29ch4_closed_delta_delta_boredom - ch4_closed_delta_delta_frustration.png]

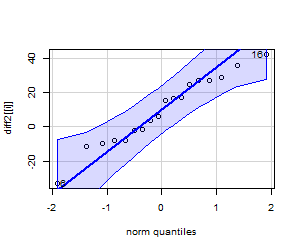

Supplement: Supplementary file 1 — Supplementary Material 1 [file 41598_2025_95647_MOESM1_ESM.zip › Supplementary/QQplot_29ch4_closed_delta_delta_flow - ch4_closed_delta_delta_frustration.png]

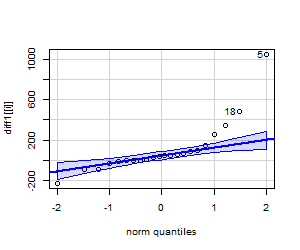

Supplement: Supplementary file 1 — Supplementary Material 1 [file 41598_2025_95647_MOESM1_ESM.zip › Supplementary/QQplot_2Acc_std_SD_boredom - Acc_std_SD_flow.png]

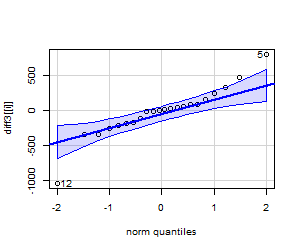

Supplement: Supplementary file 1 — Supplementary Material 1 [file 41598_2025_95647_MOESM1_ESM.zip › Supplementary/QQplot_2Acc_std_SD_boredom - Acc_std_SD_frustration.png]

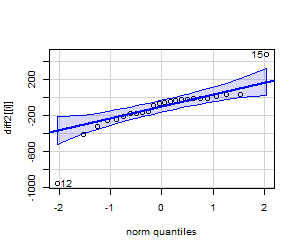

Supplement: Supplementary file 1 — Supplementary Material 1 [file 41598_2025_95647_MOESM1_ESM.zip › Supplementary/QQplot_2Acc_std_SD_flow - Acc_std_SD_frustration.png]

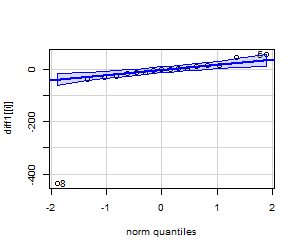

Supplement: Supplementary file 1 — Supplementary Material 1 [file 41598_2025_95647_MOESM1_ESM.zip › Supplementary/QQplot_2ch1_open_theta_theta_boredom - ch1_open_theta_theta_flow.png]

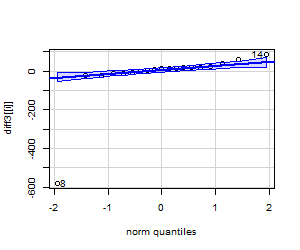

Supplement: Supplementary file 1 — Supplementary Material 1 [file 41598_2025_95647_MOESM1_ESM.zip › Supplementary/QQplot_2ch1_open_theta_theta_boredom - ch1_open_theta_theta_frustration.png]

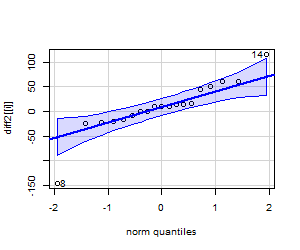

Supplement: Supplementary file 1 — Supplementary Material 1 [file 41598_2025_95647_MOESM1_ESM.zip › Supplementary/QQplot_2ch1_open_theta_theta_flow - ch1_open_theta_theta_frustration.png]

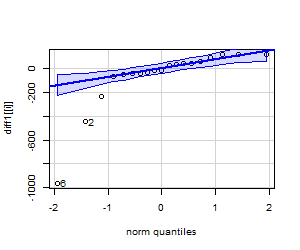

Supplement: Supplementary file 1 — Supplementary Material 1 [file 41598_2025_95647_MOESM1_ESM.zip › Supplementary/QQplot_2GSR_SD_boredom - GSR_SD_flow.png]

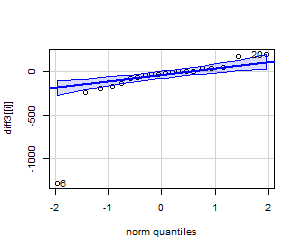

Supplement: Supplementary file 1 — Supplementary Material 1 [file 41598_2025_95647_MOESM1_ESM.zip › Supplementary/QQplot_2GSR_SD_boredom - GSR_SD_frustration.png]

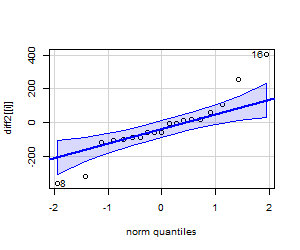

Supplement: Supplementary file 1 — Supplementary Material 1 [file 41598_2025_95647_MOESM1_ESM.zip › Supplementary/QQplot_2GSR_SD_flow - GSR_SD_frustration.png]

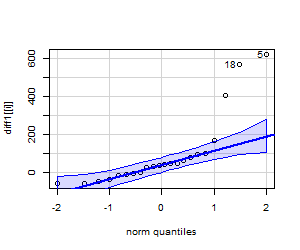

Supplement: Supplementary file 1 — Supplementary Material 1 [file 41598_2025_95647_MOESM1_ESM.zip › Supplementary/QQplot_2Gyro_std_SD_boredom - Gyro_std_SD_flow.png]

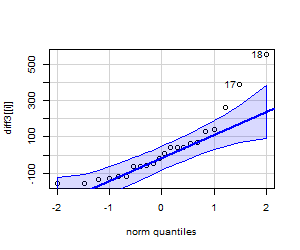

Supplement: Supplementary file 1 — Supplementary Material 1 [file 41598_2025_95647_MOESM1_ESM.zip › Supplementary/QQplot_2Gyro_std_SD_boredom - Gyro_std_SD_frustration.png]

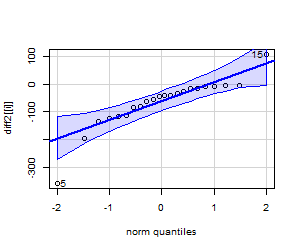

Supplement: Supplementary file 1 — Supplementary Material 1 [file 41598_2025_95647_MOESM1_ESM.zip › Supplementary/QQplot_2Gyro_std_SD_flow - Gyro_std_SD_frustration.png]

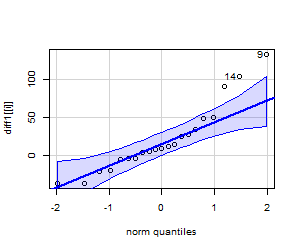

Supplement: Supplementary file 1 — Supplementary Material 1 [file 41598_2025_95647_MOESM1_ESM.zip › Supplementary/QQplot_2HR_SD_boredom - HR_SD_flow.png]

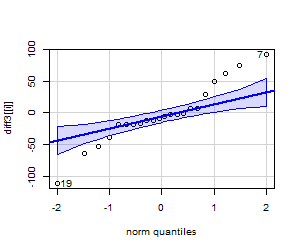

Supplement: Supplementary file 1 — Supplementary Material 1 [file 41598_2025_95647_MOESM1_ESM.zip › Supplementary/QQplot_2HR_SD_boredom - HR_SD_frustration.png]

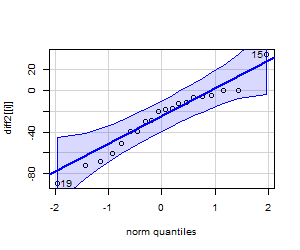

Supplement: Supplementary file 1 — Supplementary Material 1 [file 41598_2025_95647_MOESM1_ESM.zip › Supplementary/QQplot_2HR_SD_flow - HR_SD_frustration.png]

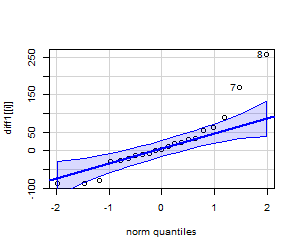

Supplement: Supplementary file 1 — Supplementary Material 1 [file 41598_2025_95647_MOESM1_ESM.zip › Supplementary/QQplot_2SPO2_SD_boredom - SPO2_SD_flow.png]

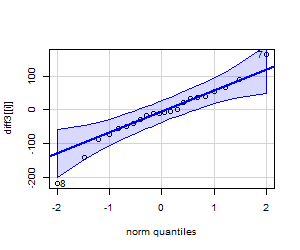

Supplement: Supplementary file 1 — Supplementary Material 1 [file 41598_2025_95647_MOESM1_ESM.zip › Supplementary/QQplot_2SPO2_SD_boredom - SPO2_SD_frustration.png]

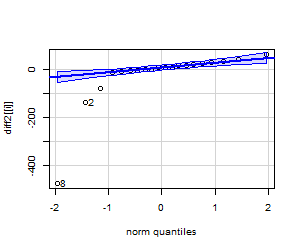

Supplement: Supplementary file 1 — Supplementary Material 1 [file 41598_2025_95647_MOESM1_ESM.zip › Supplementary/QQplot_2SPO2_SD_flow - SPO2_SD_frustration.png]

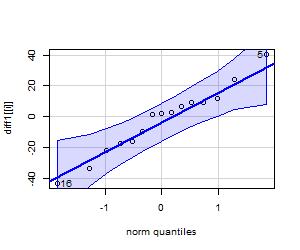

Supplement: Supplementary file 1 — Supplementary Material 1 [file 41598_2025_95647_MOESM1_ESM.zip › Supplementary/QQplot_30ch4_closed_theta_theta_boredom - ch4_closed_theta_theta_flow.png]

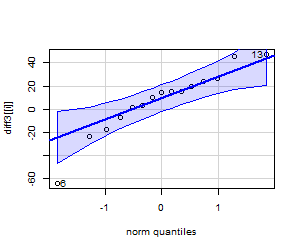

Supplement: Supplementary file 1 — Supplementary Material 1 [file 41598_2025_95647_MOESM1_ESM.zip › Supplementary/QQplot_30ch4_closed_theta_theta_boredom - ch4_closed_theta_theta_frustration.png]

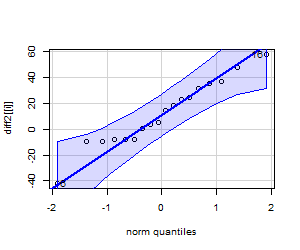

Supplement: Supplementary file 1 — Supplementary Material 1 [file 41598_2025_95647_MOESM1_ESM.zip › Supplementary/QQplot_30ch4_closed_theta_theta_flow - ch4_closed_theta_theta_frustration.png]

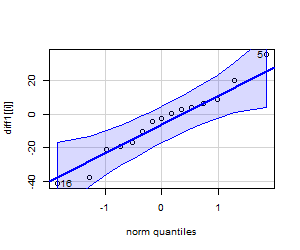

Supplement: Supplementary file 1 — Supplementary Material 1 [file 41598_2025_95647_MOESM1_ESM.zip › Supplementary/QQplot_31ch4_closed_alpha_alpha_boredom - ch4_closed_alpha_alpha_flow.png]

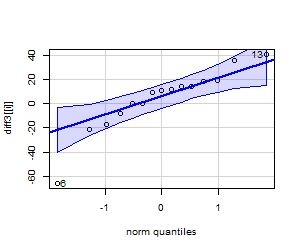

Supplement: Supplementary file 1 — Supplementary Material 1 [file 41598_2025_95647_MOESM1_ESM.zip › Supplementary/QQplot_31ch4_closed_alpha_alpha_boredom - ch4_closed_alpha_alpha_frustration.png]

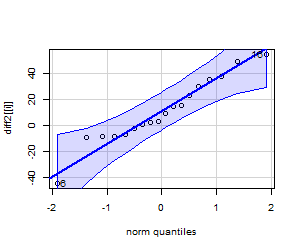

Supplement: Supplementary file 1 — Supplementary Material 1 [file 41598_2025_95647_MOESM1_ESM.zip › Supplementary/QQplot_31ch4_closed_alpha_alpha_flow - ch4_closed_alpha_alpha_frustration.png]

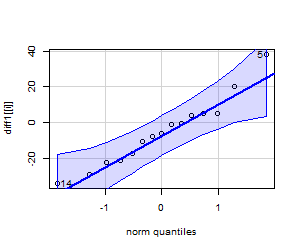

Supplement: Supplementary file 1 — Supplementary Material 1 [file 41598_2025_95647_MOESM1_ESM.zip › Supplementary/QQplot_32ch4_closed_beta_beta_boredom - ch4_closed_beta_beta_flow.png]

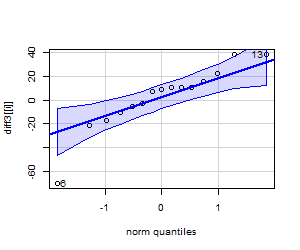

Supplement: Supplementary file 1 — Supplementary Material 1 [file 41598_2025_95647_MOESM1_ESM.zip › Supplementary/QQplot_32ch4_closed_beta_beta_boredom - ch4_closed_beta_beta_frustration.png]

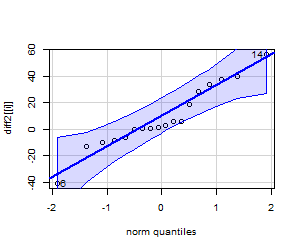

Supplement: Supplementary file 1 — Supplementary Material 1 [file 41598_2025_95647_MOESM1_ESM.zip › Supplementary/QQplot_32ch4_closed_beta_beta_flow - ch4_closed_beta_beta_frustration.png]

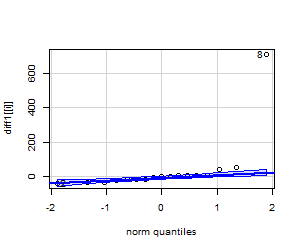

Supplement: Supplementary file 1 — Supplementary Material 1 [file 41598_2025_95647_MOESM1_ESM.zip › Supplementary/QQplot_3ch1_open_alpha_alpha_boredom - ch1_open_alpha_alpha_flow.png]

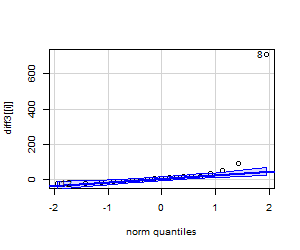

Supplement: Supplementary file 1 — Supplementary Material 1 [file 41598_2025_95647_MOESM1_ESM.zip › Supplementary/QQplot_3ch1_open_alpha_alpha_boredom - ch1_open_alpha_alpha_frustration.png]

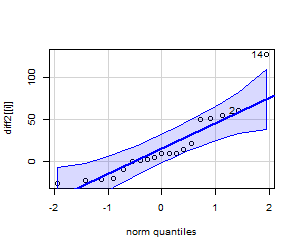

Supplement: Supplementary file 1 — Supplementary Material 1 [file 41598_2025_95647_MOESM1_ESM.zip › Supplementary/QQplot_3ch1_open_alpha_alpha_flow - ch1_open_alpha_alpha_frustration.png]

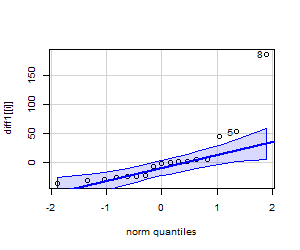

Supplement: Supplementary file 1 — Supplementary Material 1 [file 41598_2025_95647_MOESM1_ESM.zip › Supplementary/QQplot_4ch1_open_beta_beta_boredom - ch1_open_beta_beta_flow.png]

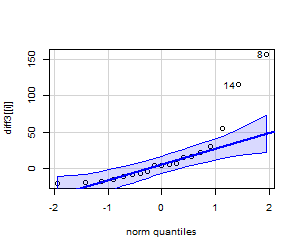

Supplement: Supplementary file 1 — Supplementary Material 1 [file 41598_2025_95647_MOESM1_ESM.zip › Supplementary/QQplot_4ch1_open_beta_beta_boredom - ch1_open_beta_beta_frustration.png]

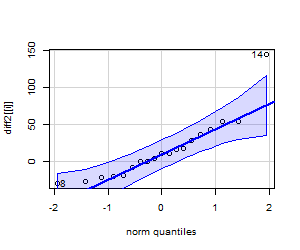

Supplement: Supplementary file 1 — Supplementary Material 1 [file 41598_2025_95647_MOESM1_ESM.zip › Supplementary/QQplot_4ch1_open_beta_beta_flow - ch1_open_beta_beta_frustration.png]

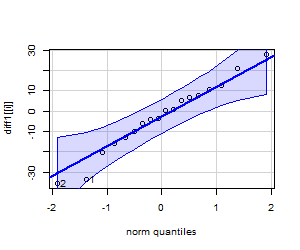

Supplement: Supplementary file 1 — Supplementary Material 1 [file 41598_2025_95647_MOESM1_ESM.zip › Supplementary/QQplot_5ch2_open_delta_delta_boredom - ch2_open_delta_delta_flow.png]

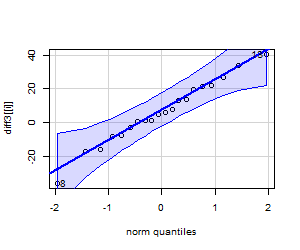

Supplement: Supplementary file 1 — Supplementary Material 1 [file 41598_2025_95647_MOESM1_ESM.zip › Supplementary/QQplot_5ch2_open_delta_delta_boredom - ch2_open_delta_delta_frustration.png]

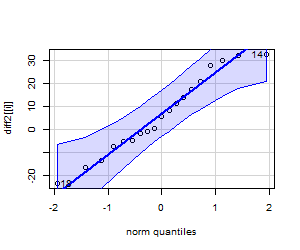

Supplement: Supplementary file 1 — Supplementary Material 1 [file 41598_2025_95647_MOESM1_ESM.zip › Supplementary/QQplot_5ch2_open_delta_delta_flow - ch2_open_delta_delta_frustration.png]

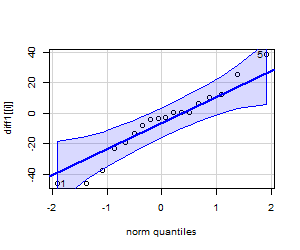

Supplement: Supplementary file 1 — Supplementary Material 1 [file 41598_2025_95647_MOESM1_ESM.zip › Supplementary/QQplot_6ch2_open_theta_theta_boredom - ch2_open_theta_theta_flow.png]

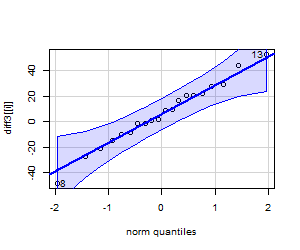

Supplement: Supplementary file 1 — Supplementary Material 1 [file 41598_2025_95647_MOESM1_ESM.zip › Supplementary/QQplot_6ch2_open_theta_theta_boredom - ch2_open_theta_theta_frustration.png]

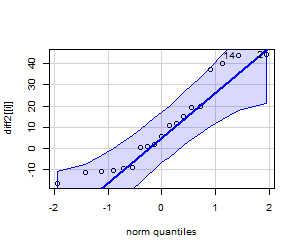

Supplement: Supplementary file 1 — Supplementary Material 1 [file 41598_2025_95647_MOESM1_ESM.zip › Supplementary/QQplot_6ch2_open_theta_theta_flow - ch2_open_theta_theta_frustration.png]

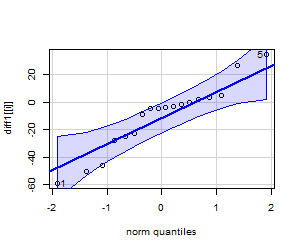

Supplement: Supplementary file 1 — Supplementary Material 1 [file 41598_2025_95647_MOESM1_ESM.zip › Supplementary/QQplot_7ch2_open_alpha_alpha_boredom - ch2_open_alpha_alpha_flow.png]

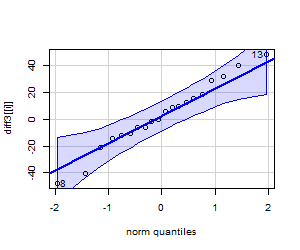

Supplement: Supplementary file 1 — Supplementary Material 1 [file 41598_2025_95647_MOESM1_ESM.zip › Supplementary/QQplot_7ch2_open_alpha_alpha_boredom - ch2_open_alpha_alpha_frustration.png]

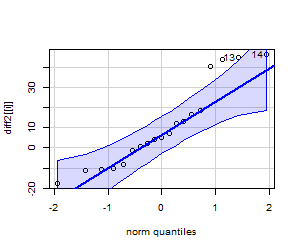

Supplement: Supplementary file 1 — Supplementary Material 1 [file 41598_2025_95647_MOESM1_ESM.zip › Supplementary/QQplot_7ch2_open_alpha_alpha_flow - ch2_open_alpha_alpha_frustration.png]

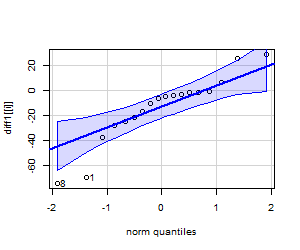

Supplement: Supplementary file 1 — Supplementary Material 1 [file 41598_2025_95647_MOESM1_ESM.zip › Supplementary/QQplot_8ch2_open_beta_beta_boredom - ch2_open_beta_beta_flow.png]

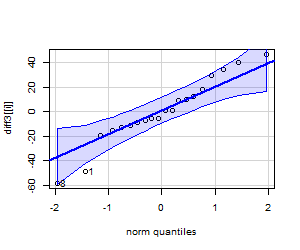

Supplement: Supplementary file 1 — Supplementary Material 1 [file 41598_2025_95647_MOESM1_ESM.zip › Supplementary/QQplot_8ch2_open_beta_beta_boredom - ch2_open_beta_beta_frustration.png]

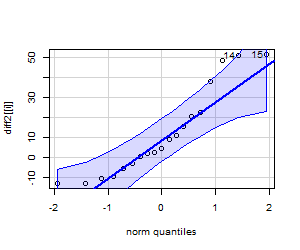

Supplement: Supplementary file 1 — Supplementary Material 1 [file 41598_2025_95647_MOESM1_ESM.zip › Supplementary/QQplot_8ch2_open_beta_beta_flow - ch2_open_beta_beta_frustration.png]

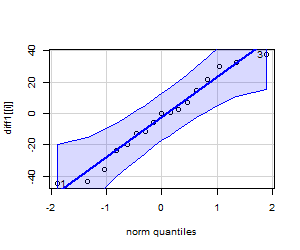

Supplement: Supplementary file 1 — Supplementary Material 1 [file 41598_2025_95647_MOESM1_ESM.zip › Supplementary/QQplot_9ch3_open_delta_delta_boredom - ch3_open_delta_delta_flow.png]

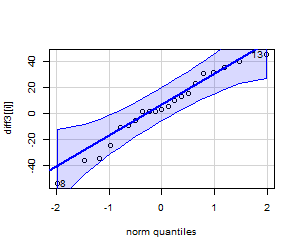

Supplement: Supplementary file 1 — Supplementary Material 1 [file 41598_2025_95647_MOESM1_ESM.zip › Supplementary/QQplot_9ch3_open_delta_delta_boredom - ch3_open_delta_delta_frustration.png]

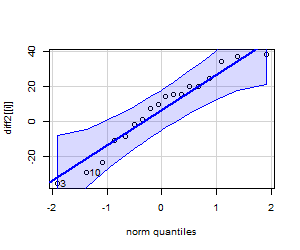

Supplement: Supplementary file 1 — Supplementary Material 1 [file 41598_2025_95647_MOESM1_ESM.zip › Supplementary/QQplot_9ch3_open_delta_delta_flow - ch3_open_delta_delta_frustration.png]
