# Supplementary figures and images for: Physiological assessment of the psychological flow state using wearable devices (part 1 of 2)
Source: Sci Rep. 2025 Apr 7;15:11839. doi: 10.1038/s41598-025-95647-x (PMC11977251; doi:10.1038/s41598-025-95647-x)

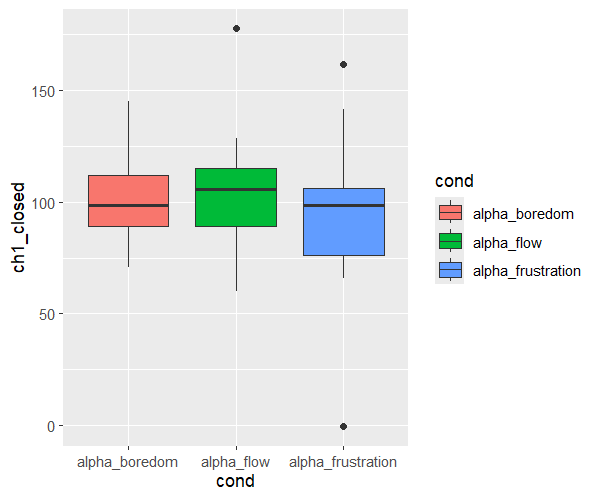

Supplement: Supplementary file 1 — Supplementary Material 1 [file 41598_2025_95647_MOESM1_ESM.zip › Supplementary/boxplot_alpha_ch1_closed.png]

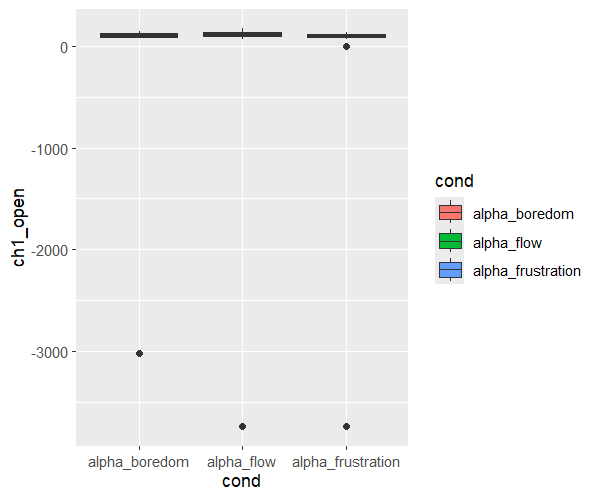

Supplement: Supplementary file 1 — Supplementary Material 1 [file 41598_2025_95647_MOESM1_ESM.zip › Supplementary/boxplot_alpha_ch1_open.png]

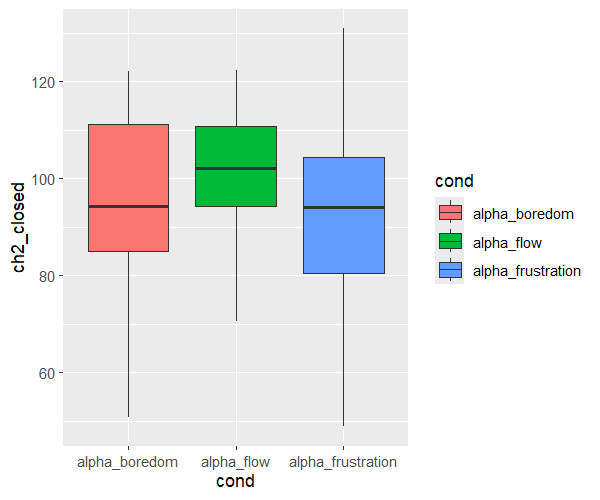

Supplement: Supplementary file 1 — Supplementary Material 1 [file 41598_2025_95647_MOESM1_ESM.zip › Supplementary/boxplot_alpha_ch2_closed.png]

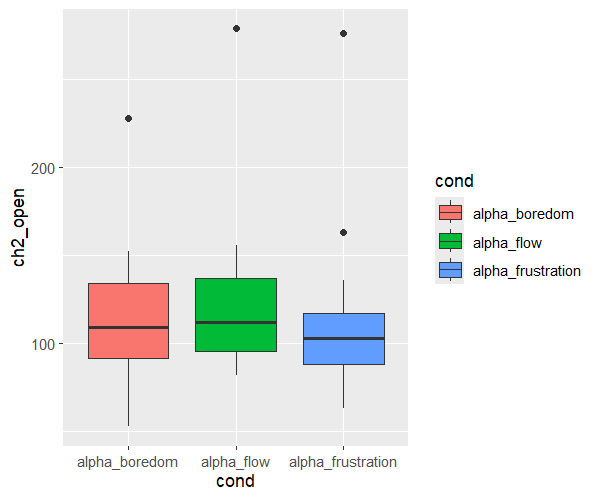

Supplement: Supplementary file 1 — Supplementary Material 1 [file 41598_2025_95647_MOESM1_ESM.zip › Supplementary/boxplot_alpha_ch2_open.png]

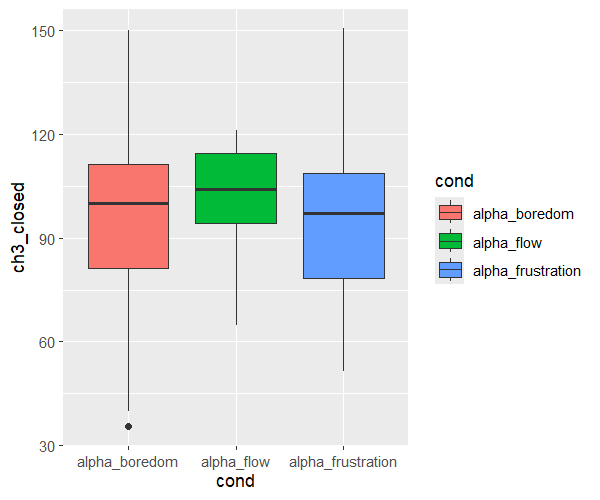

Supplement: Supplementary file 1 — Supplementary Material 1 [file 41598_2025_95647_MOESM1_ESM.zip › Supplementary/boxplot_alpha_ch3_closed.png]

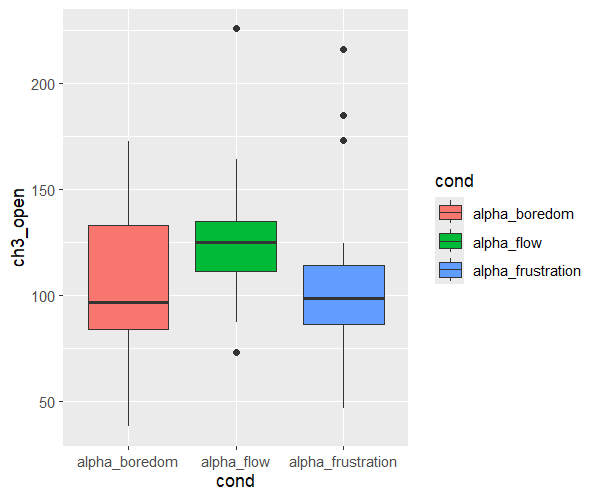

Supplement: Supplementary file 1 — Supplementary Material 1 [file 41598_2025_95647_MOESM1_ESM.zip › Supplementary/boxplot_alpha_ch3_open.png]

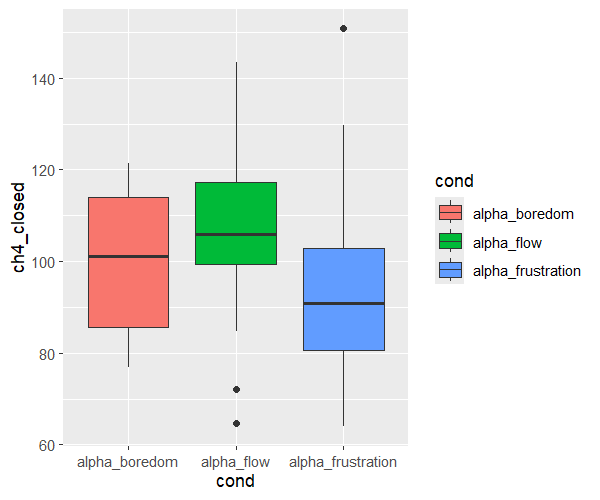

Supplement: Supplementary file 1 — Supplementary Material 1 [file 41598_2025_95647_MOESM1_ESM.zip › Supplementary/boxplot_alpha_ch4_closed.png]

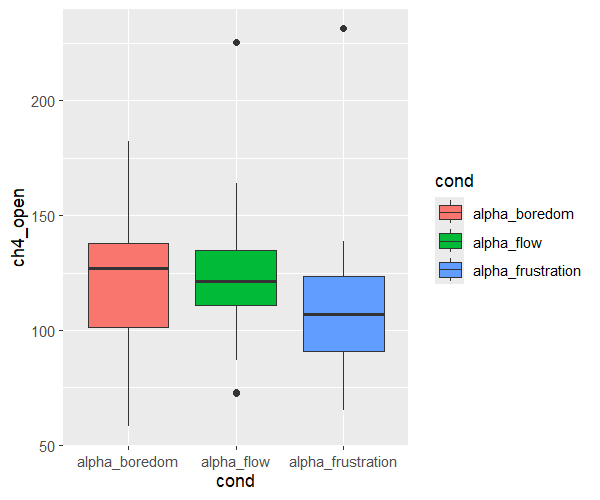

Supplement: Supplementary file 1 — Supplementary Material 1 [file 41598_2025_95647_MOESM1_ESM.zip › Supplementary/boxplot_alpha_ch4_open.png]

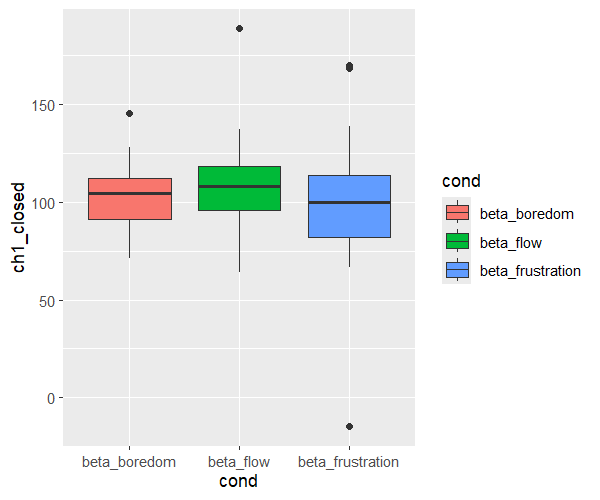

Supplement: Supplementary file 1 — Supplementary Material 1 [file 41598_2025_95647_MOESM1_ESM.zip › Supplementary/boxplot_beta_ch1_closed.png]

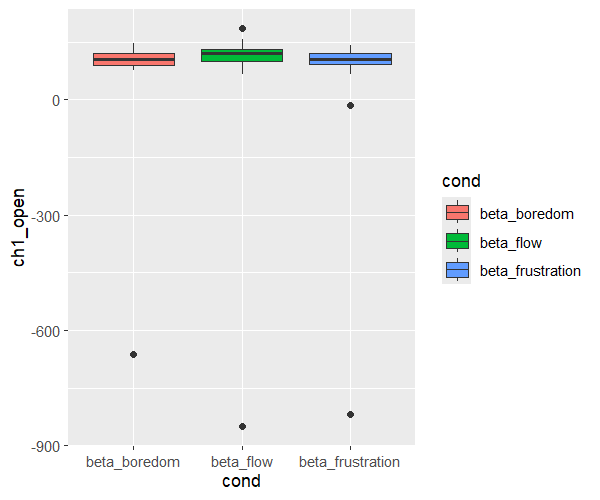

Supplement: Supplementary file 1 — Supplementary Material 1 [file 41598_2025_95647_MOESM1_ESM.zip › Supplementary/boxplot_beta_ch1_open.png]

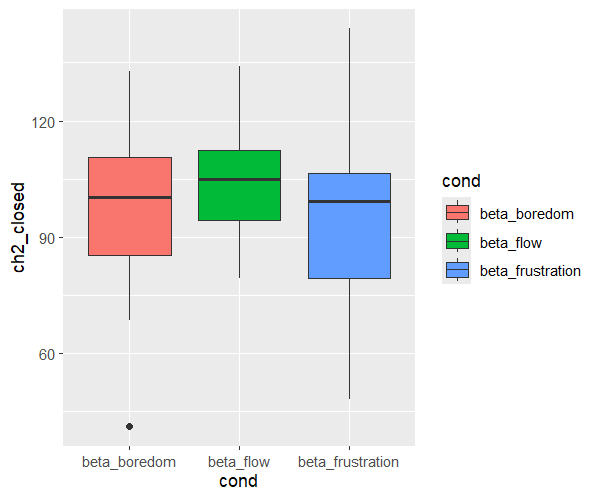

Supplement: Supplementary file 1 — Supplementary Material 1 [file 41598_2025_95647_MOESM1_ESM.zip › Supplementary/boxplot_beta_ch2_closed.png]

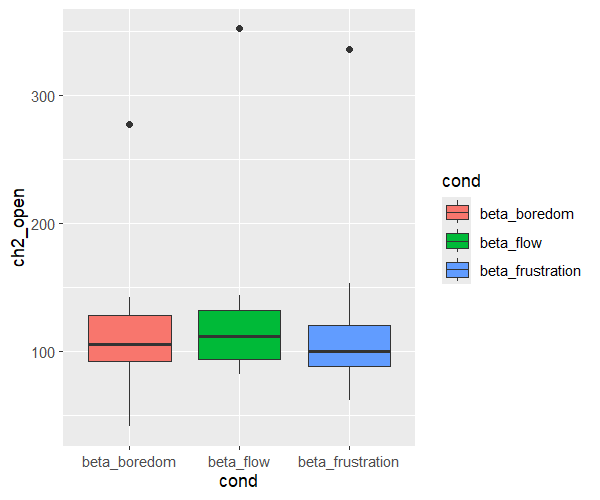

Supplement: Supplementary file 1 — Supplementary Material 1 [file 41598_2025_95647_MOESM1_ESM.zip › Supplementary/boxplot_beta_ch2_open.png]

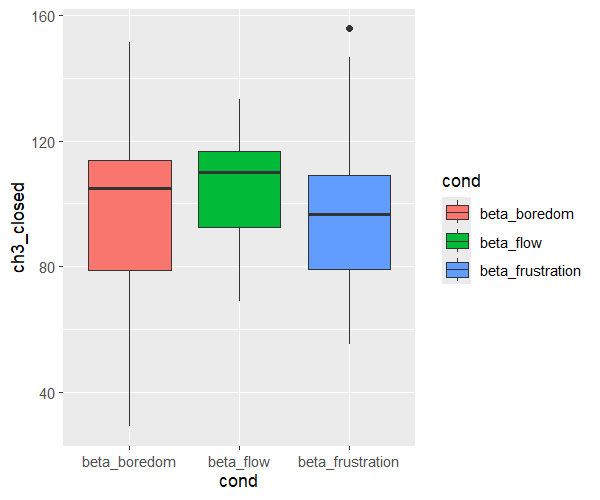

Supplement: Supplementary file 1 — Supplementary Material 1 [file 41598_2025_95647_MOESM1_ESM.zip › Supplementary/boxplot_beta_ch3_closed.png]

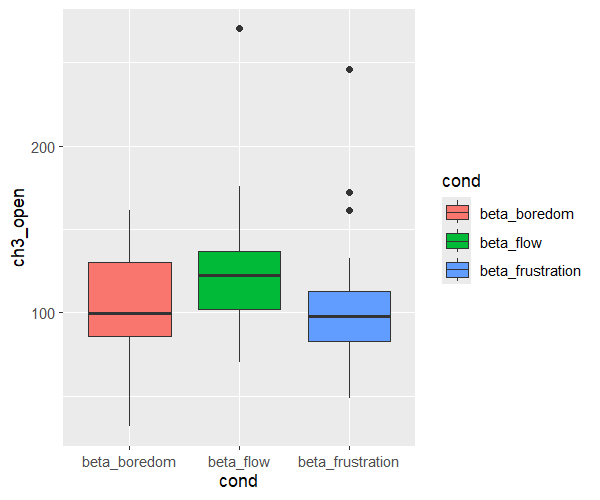

Supplement: Supplementary file 1 — Supplementary Material 1 [file 41598_2025_95647_MOESM1_ESM.zip › Supplementary/boxplot_beta_ch3_open.png]

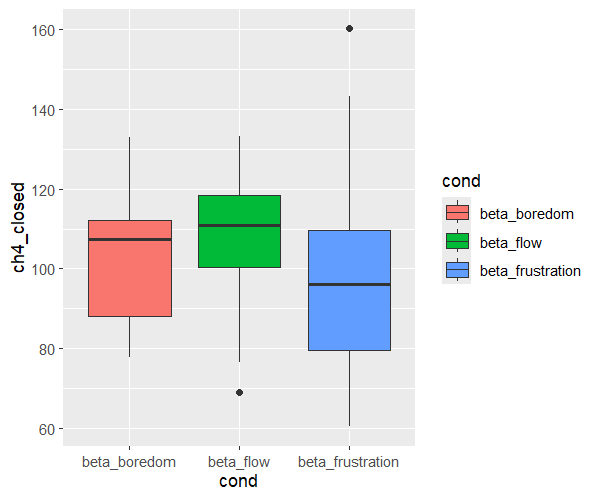

Supplement: Supplementary file 1 — Supplementary Material 1 [file 41598_2025_95647_MOESM1_ESM.zip › Supplementary/boxplot_beta_ch4_closed.png]

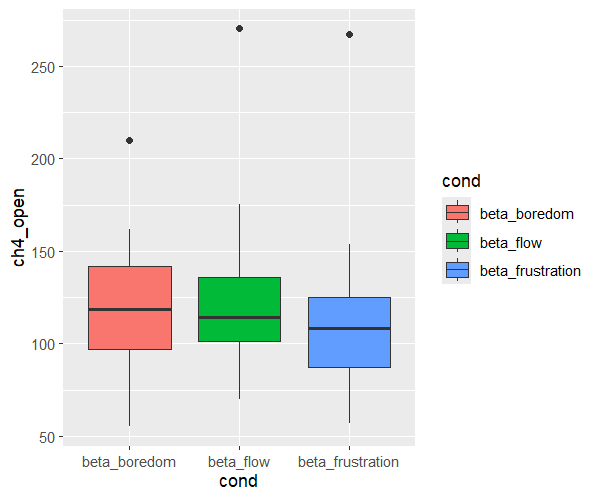

Supplement: Supplementary file 1 — Supplementary Material 1 [file 41598_2025_95647_MOESM1_ESM.zip › Supplementary/boxplot_beta_ch4_open.png]

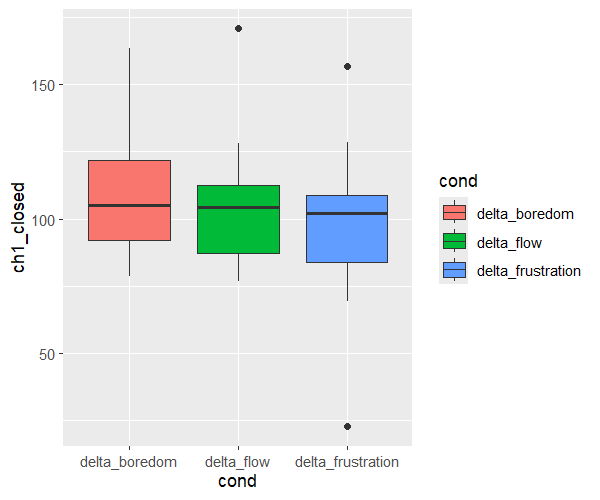

Supplement: Supplementary file 1 — Supplementary Material 1 [file 41598_2025_95647_MOESM1_ESM.zip › Supplementary/boxplot_delta_ch1_closed.png]

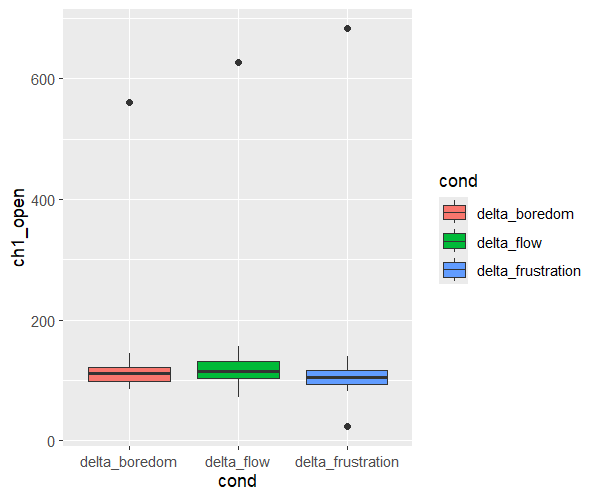

Supplement: Supplementary file 1 — Supplementary Material 1 [file 41598_2025_95647_MOESM1_ESM.zip › Supplementary/boxplot_delta_ch1_open.png]

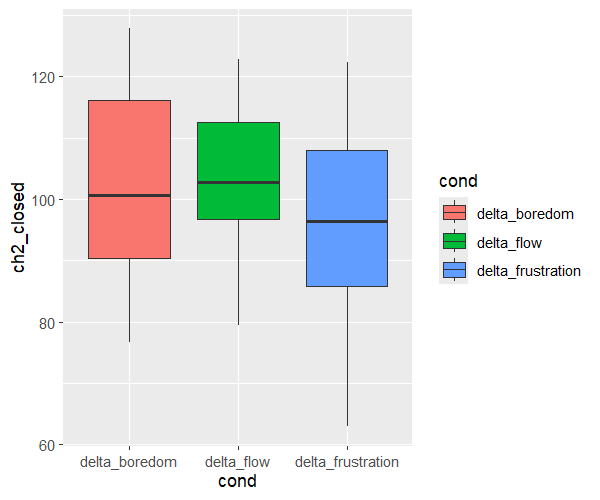

Supplement: Supplementary file 1 — Supplementary Material 1 [file 41598_2025_95647_MOESM1_ESM.zip › Supplementary/boxplot_delta_ch2_closed.png]

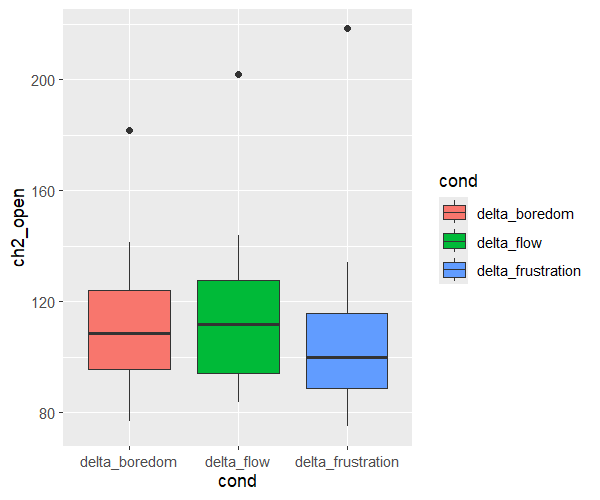

Supplement: Supplementary file 1 — Supplementary Material 1 [file 41598_2025_95647_MOESM1_ESM.zip › Supplementary/boxplot_delta_ch2_open.png]

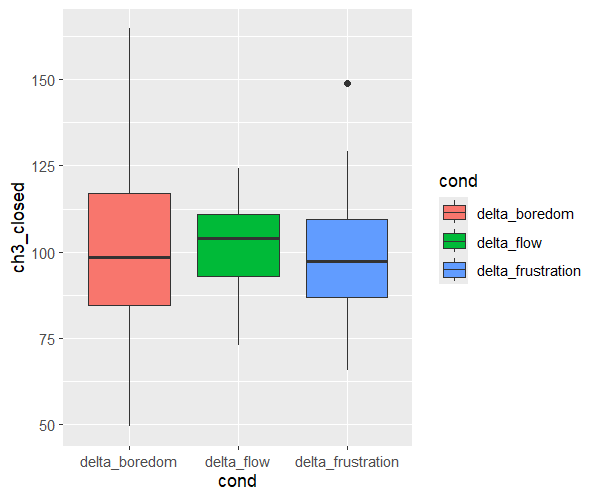

Supplement: Supplementary file 1 — Supplementary Material 1 [file 41598_2025_95647_MOESM1_ESM.zip › Supplementary/boxplot_delta_ch3_closed.png]

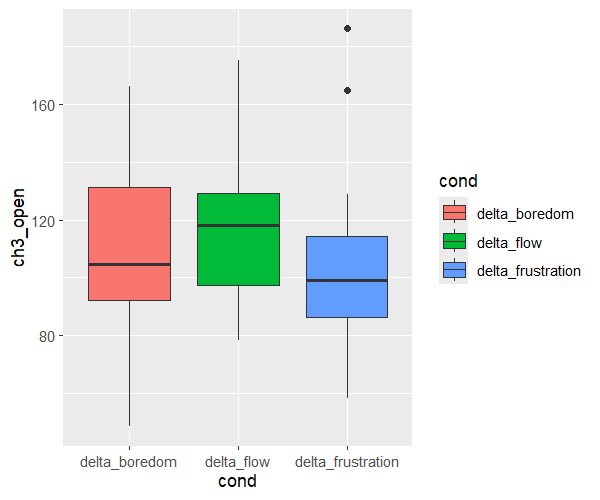

Supplement: Supplementary file 1 — Supplementary Material 1 [file 41598_2025_95647_MOESM1_ESM.zip › Supplementary/boxplot_delta_ch3_open.png]

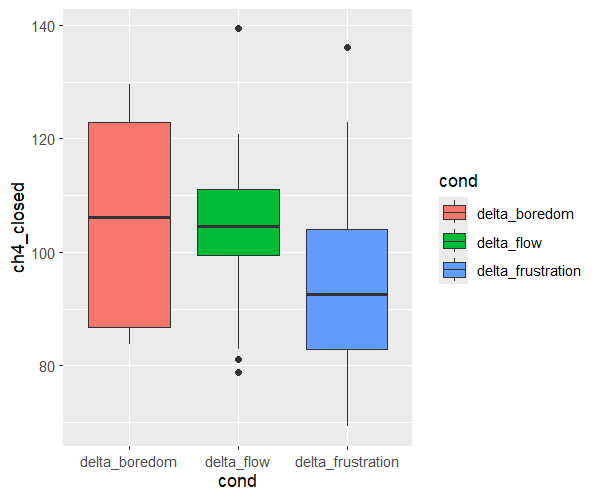

Supplement: Supplementary file 1 — Supplementary Material 1 [file 41598_2025_95647_MOESM1_ESM.zip › Supplementary/boxplot_delta_ch4_closed.png]

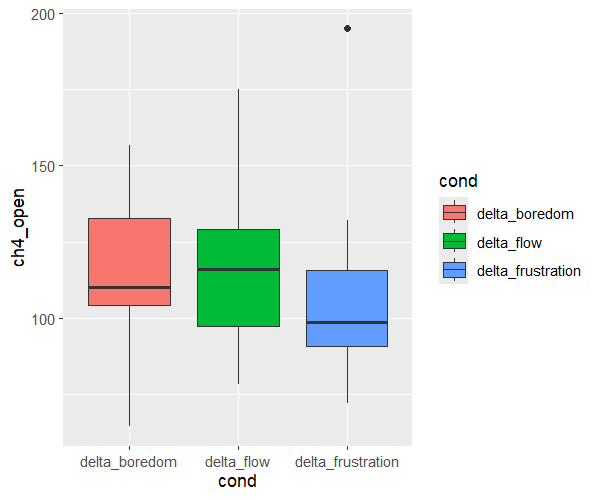

Supplement: Supplementary file 1 — Supplementary Material 1 [file 41598_2025_95647_MOESM1_ESM.zip › Supplementary/boxplot_delta_ch4_open.png]

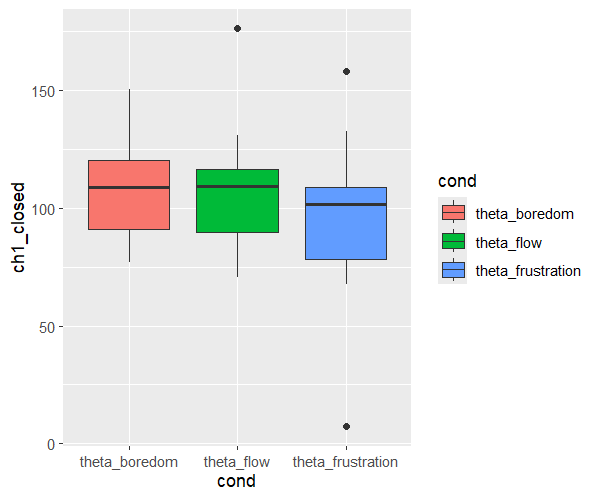

Supplement: Supplementary file 1 — Supplementary Material 1 [file 41598_2025_95647_MOESM1_ESM.zip › Supplementary/boxplot_theta_ch1_closed.png]

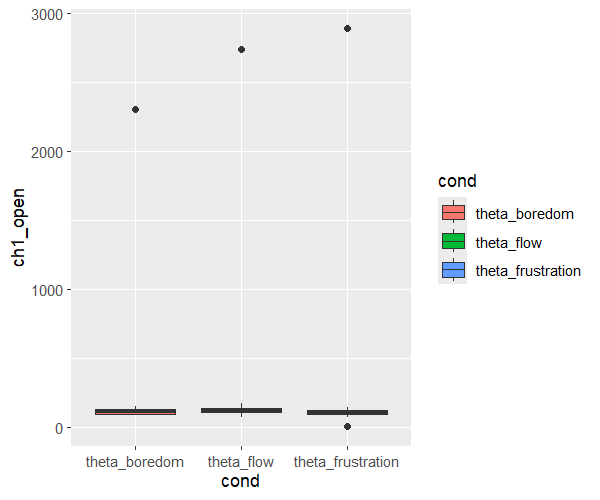

Supplement: Supplementary file 1 — Supplementary Material 1 [file 41598_2025_95647_MOESM1_ESM.zip › Supplementary/boxplot_theta_ch1_open.png]

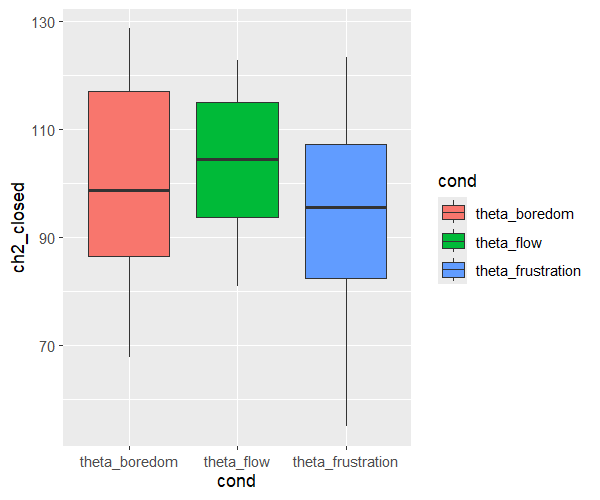

Supplement: Supplementary file 1 — Supplementary Material 1 [file 41598_2025_95647_MOESM1_ESM.zip › Supplementary/boxplot_theta_ch2_closed.png]

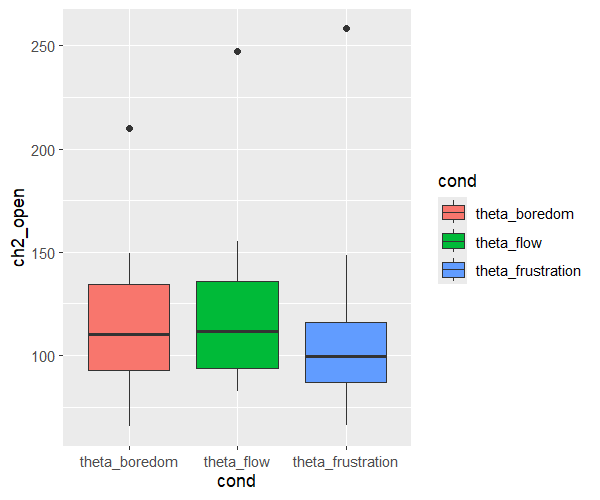

Supplement: Supplementary file 1 — Supplementary Material 1 [file 41598_2025_95647_MOESM1_ESM.zip › Supplementary/boxplot_theta_ch2_open.png]

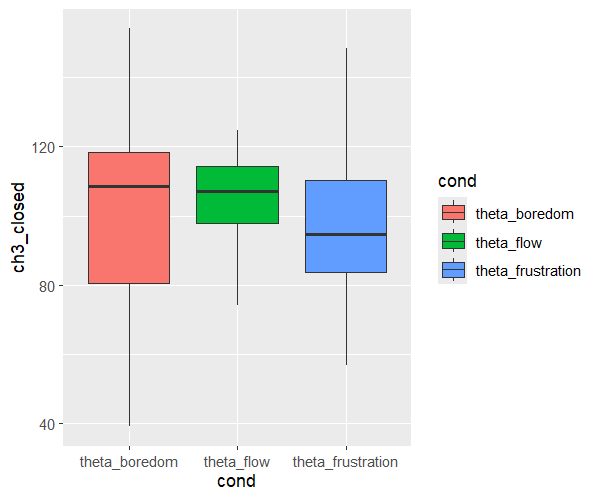

Supplement: Supplementary file 1 — Supplementary Material 1 [file 41598_2025_95647_MOESM1_ESM.zip › Supplementary/boxplot_theta_ch3_closed.png]

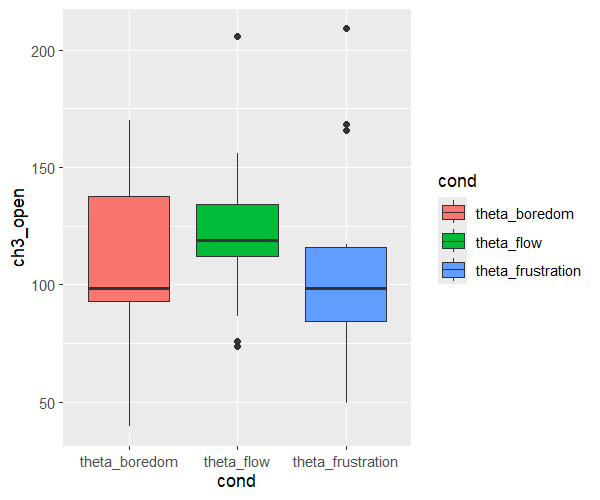

Supplement: Supplementary file 1 — Supplementary Material 1 [file 41598_2025_95647_MOESM1_ESM.zip › Supplementary/boxplot_theta_ch3_open.png]

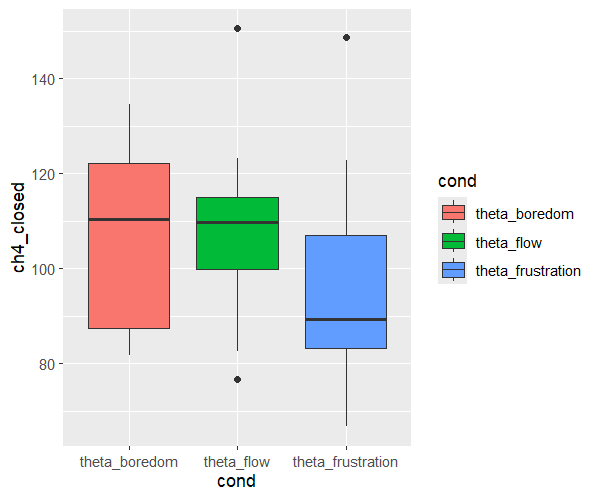

Supplement: Supplementary file 1 — Supplementary Material 1 [file 41598_2025_95647_MOESM1_ESM.zip › Supplementary/boxplot_theta_ch4_closed.png]

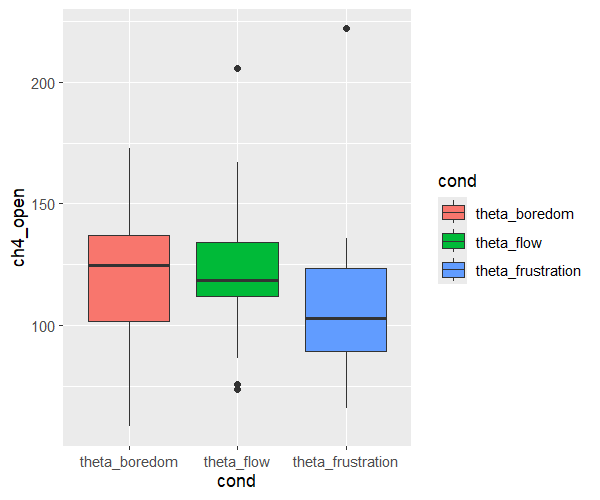

Supplement: Supplementary file 1 — Supplementary Material 1 [file 41598_2025_95647_MOESM1_ESM.zip › Supplementary/boxplot_theta_ch4_open.png]

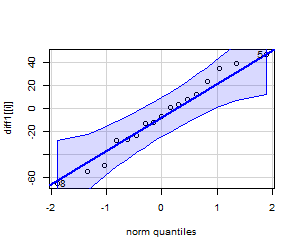

Supplement: Supplementary file 1 — Supplementary Material 1 [file 41598_2025_95647_MOESM1_ESM.zip › Supplementary/QQplot_10ch3_open_theta_theta_boredom - ch3_open_theta_theta_flow.png]

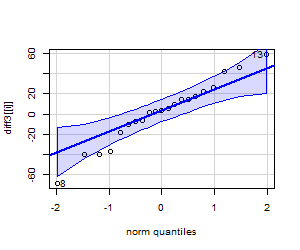

Supplement: Supplementary file 1 — Supplementary Material 1 [file 41598_2025_95647_MOESM1_ESM.zip › Supplementary/QQplot_10ch3_open_theta_theta_boredom - ch3_open_theta_theta_frustration.png]

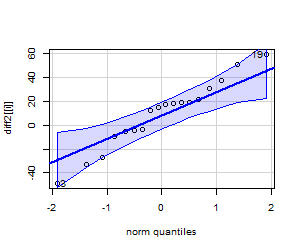

Supplement: Supplementary file 1 — Supplementary Material 1 [file 41598_2025_95647_MOESM1_ESM.zip › Supplementary/QQplot_10ch3_open_theta_theta_flow - ch3_open_theta_theta_frustration.png]

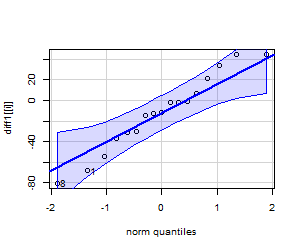

Supplement: Supplementary file 1 — Supplementary Material 1 [file 41598_2025_95647_MOESM1_ESM.zip › Supplementary/QQplot_11ch3_open_alpha_alpha_boredom - ch3_open_alpha_alpha_flow.png]

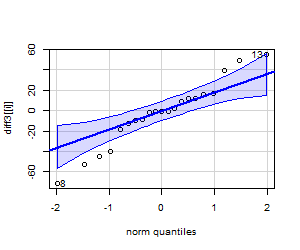

Supplement: Supplementary file 1 — Supplementary Material 1 [file 41598_2025_95647_MOESM1_ESM.zip › Supplementary/QQplot_11ch3_open_alpha_alpha_boredom - ch3_open_alpha_alpha_frustration.png]

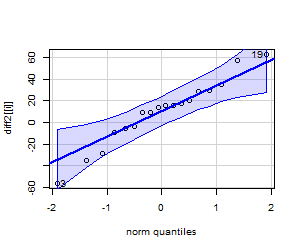

Supplement: Supplementary file 1 — Supplementary Material 1 [file 41598_2025_95647_MOESM1_ESM.zip › Supplementary/QQplot_11ch3_open_alpha_alpha_flow - ch3_open_alpha_alpha_frustration.png]

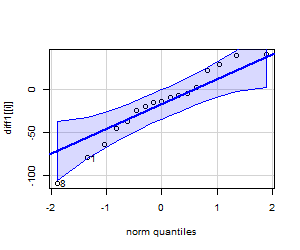

Supplement: Supplementary file 1 — Supplementary Material 1 [file 41598_2025_95647_MOESM1_ESM.zip › Supplementary/QQplot_12ch3_open_beta_beta_boredom - ch3_open_beta_beta_flow.png]

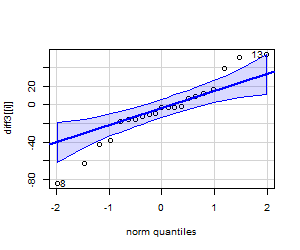

Supplement: Supplementary file 1 — Supplementary Material 1 [file 41598_2025_95647_MOESM1_ESM.zip › Supplementary/QQplot_12ch3_open_beta_beta_boredom - ch3_open_beta_beta_frustration.png]

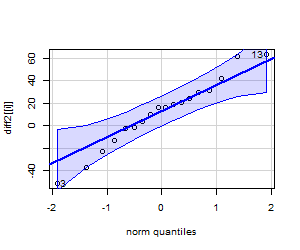

Supplement: Supplementary file 1 — Supplementary Material 1 [file 41598_2025_95647_MOESM1_ESM.zip › Supplementary/QQplot_12ch3_open_beta_beta_flow - ch3_open_beta_beta_frustration.png]

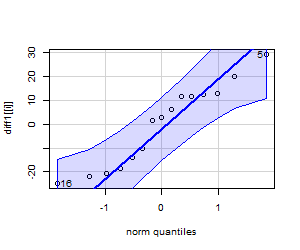

Supplement: Supplementary file 1 — Supplementary Material 1 [file 41598_2025_95647_MOESM1_ESM.zip › Supplementary/QQplot_13ch4_open_delta_delta_boredom - ch4_open_delta_delta_flow.png]

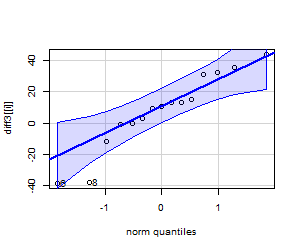

Supplement: Supplementary file 1 — Supplementary Material 1 [file 41598_2025_95647_MOESM1_ESM.zip › Supplementary/QQplot_13ch4_open_delta_delta_boredom - ch4_open_delta_delta_frustration.png]

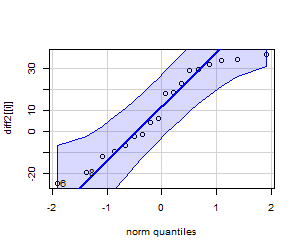

Supplement: Supplementary file 1 — Supplementary Material 1 [file 41598_2025_95647_MOESM1_ESM.zip › Supplementary/QQplot_13ch4_open_delta_delta_flow - ch4_open_delta_delta_frustration.png]

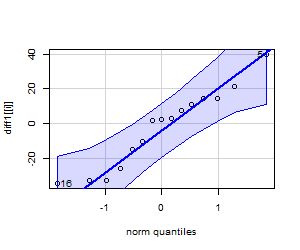

Supplement: Supplementary file 1 — Supplementary Material 1 [file 41598_2025_95647_MOESM1_ESM.zip › Supplementary/QQplot_14ch4_open_theta_theta_boredom - ch4_open_theta_theta_flow.png]

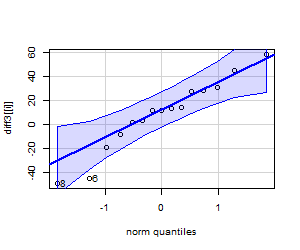

Supplement: Supplementary file 1 — Supplementary Material 1 [file 41598_2025_95647_MOESM1_ESM.zip › Supplementary/QQplot_14ch4_open_theta_theta_boredom - ch4_open_theta_theta_frustration.png]

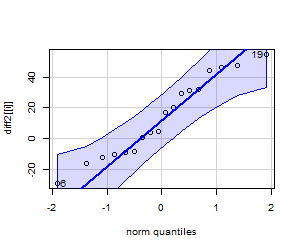

Supplement: Supplementary file 1 — Supplementary Material 1 [file 41598_2025_95647_MOESM1_ESM.zip › Supplementary/QQplot_14ch4_open_theta_theta_flow - ch4_open_theta_theta_frustration.png]

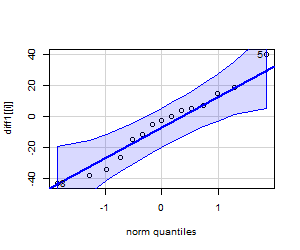

Supplement: Supplementary file 1 — Supplementary Material 1 [file 41598_2025_95647_MOESM1_ESM.zip › Supplementary/QQplot_15ch4_open_alpha_alpha_boredom - ch4_open_alpha_alpha_flow.png]

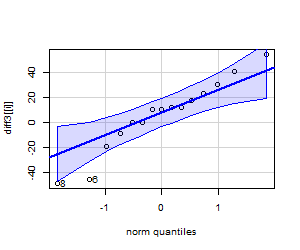

Supplement: Supplementary file 1 — Supplementary Material 1 [file 41598_2025_95647_MOESM1_ESM.zip › Supplementary/QQplot_15ch4_open_alpha_alpha_boredom - ch4_open_alpha_alpha_frustration.png]

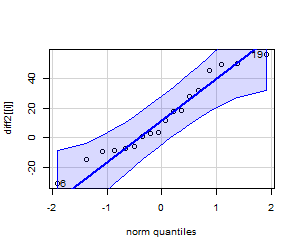

Supplement: Supplementary file 1 — Supplementary Material 1 [file 41598_2025_95647_MOESM1_ESM.zip › Supplementary/QQplot_15ch4_open_alpha_alpha_flow - ch4_open_alpha_alpha_frustration.png]

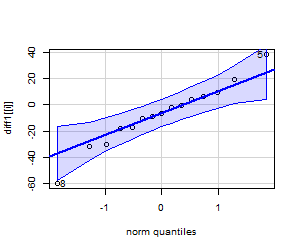

Supplement: Supplementary file 1 — Supplementary Material 1 [file 41598_2025_95647_MOESM1_ESM.zip › Supplementary/QQplot_16ch4_open_beta_beta_boredom - ch4_open_beta_beta_flow.png]

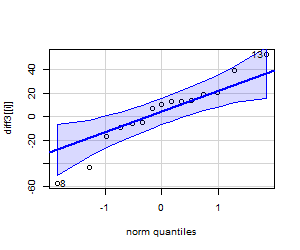

Supplement: Supplementary file 1 — Supplementary Material 1 [file 41598_2025_95647_MOESM1_ESM.zip › Supplementary/QQplot_16ch4_open_beta_beta_boredom - ch4_open_beta_beta_frustration.png]

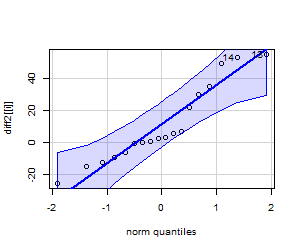

Supplement: Supplementary file 1 — Supplementary Material 1 [file 41598_2025_95647_MOESM1_ESM.zip › Supplementary/QQplot_16ch4_open_beta_beta_flow - ch4_open_beta_beta_frustration.png]

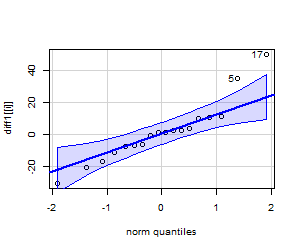

Supplement: Supplementary file 1 — Supplementary Material 1 [file 41598_2025_95647_MOESM1_ESM.zip › Supplementary/QQplot_17ch1_closed_delta_delta_boredom - ch1_closed_delta_delta_flow.png]

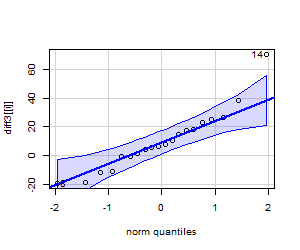

Supplement: Supplementary file 1 — Supplementary Material 1 [file 41598_2025_95647_MOESM1_ESM.zip › Supplementary/QQplot_17ch1_closed_delta_delta_boredom - ch1_closed_delta_delta_frustration.png]

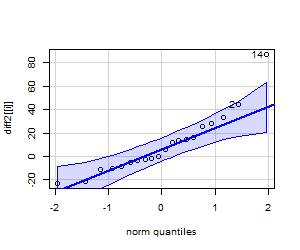

Supplement: Supplementary file 1 — Supplementary Material 1 [file 41598_2025_95647_MOESM1_ESM.zip › Supplementary/QQplot_17ch1_closed_delta_delta_flow - ch1_closed_delta_delta_frustration.png]

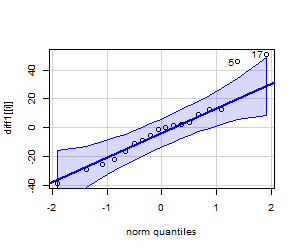

Supplement: Supplementary file 1 — Supplementary Material 1 [file 41598_2025_95647_MOESM1_ESM.zip › Supplementary/QQplot_18ch1_closed_theta_theta_boredom - ch1_closed_theta_theta_flow.png]

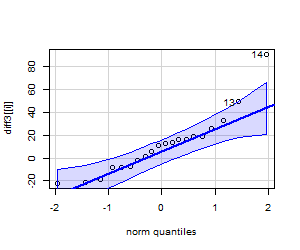

Supplement: Supplementary file 1 — Supplementary Material 1 [file 41598_2025_95647_MOESM1_ESM.zip › Supplementary/QQplot_18ch1_closed_theta_theta_boredom - ch1_closed_theta_theta_frustration.png]

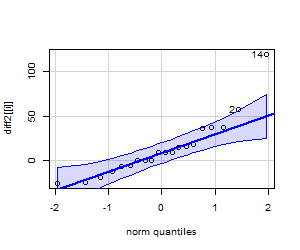

Supplement: Supplementary file 1 — Supplementary Material 1 [file 41598_2025_95647_MOESM1_ESM.zip › Supplementary/QQplot_18ch1_closed_theta_theta_flow - ch1_closed_theta_theta_frustration.png]

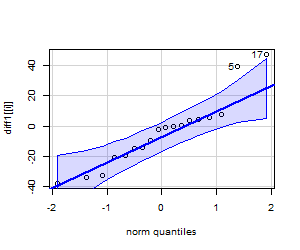

Supplement: Supplementary file 1 — Supplementary Material 1 [file 41598_2025_95647_MOESM1_ESM.zip › Supplementary/QQplot_19ch1_closed_alpha_alpha_boredom - ch1_closed_alpha_alpha_flow.png]

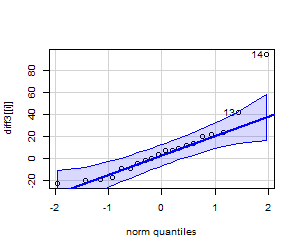

Supplement: Supplementary file 1 — Supplementary Material 1 [file 41598_2025_95647_MOESM1_ESM.zip › Supplementary/QQplot_19ch1_closed_alpha_alpha_boredom - ch1_closed_alpha_alpha_frustration.png]

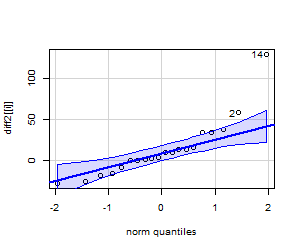

Supplement: Supplementary file 1 — Supplementary Material 1 [file 41598_2025_95647_MOESM1_ESM.zip › Supplementary/QQplot_19ch1_closed_alpha_alpha_flow - ch1_closed_alpha_alpha_frustration.png]

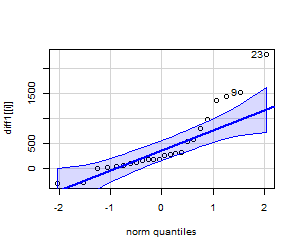

Supplement: Supplementary file 1 — Supplementary Material 1 [file 41598_2025_95647_MOESM1_ESM.zip › Supplementary/QQplot_1Acc_std_boredom - Acc_std_flow.png]

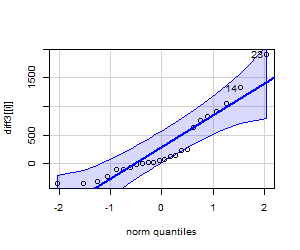

Supplement: Supplementary file 1 — Supplementary Material 1 [file 41598_2025_95647_MOESM1_ESM.zip › Supplementary/QQplot_1Acc_std_boredom - Acc_std_frustration.png]

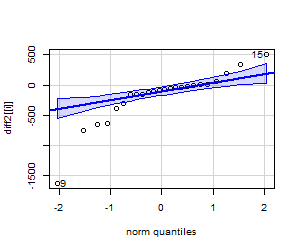

Supplement: Supplementary file 1 — Supplementary Material 1 [file 41598_2025_95647_MOESM1_ESM.zip › Supplementary/QQplot_1Acc_std_flow - Acc_std_frustration.png]

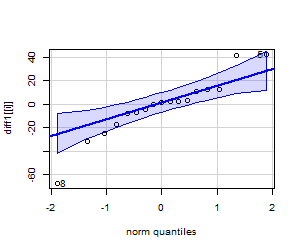

Supplement: Supplementary file 1 — Supplementary Material 1 [file 41598_2025_95647_MOESM1_ESM.zip › Supplementary/QQplot_1ch1_open_delta_delta_boredom - ch1_open_delta_delta_flow.png]

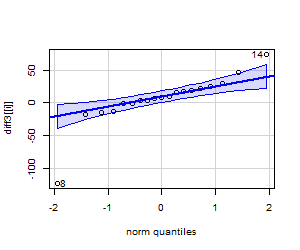

Supplement: Supplementary file 1 — Supplementary Material 1 [file 41598_2025_95647_MOESM1_ESM.zip › Supplementary/QQplot_1ch1_open_delta_delta_boredom - ch1_open_delta_delta_frustration.png]

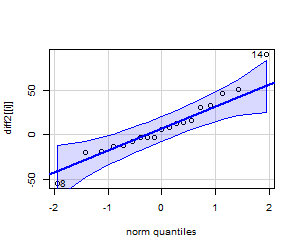

Supplement: Supplementary file 1 — Supplementary Material 1 [file 41598_2025_95647_MOESM1_ESM.zip › Supplementary/QQplot_1ch1_open_delta_delta_flow - ch1_open_delta_delta_frustration.png]

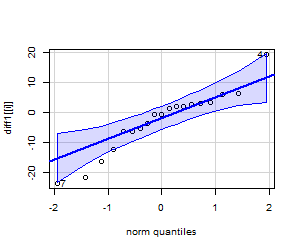

Supplement: Supplementary file 1 — Supplementary Material 1 [file 41598_2025_95647_MOESM1_ESM.zip › Supplementary/QQplot_1GSR_boredom - GSR_flow.png]

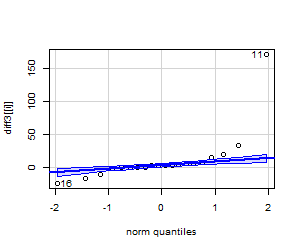

Supplement: Supplementary file 1 — Supplementary Material 1 [file 41598_2025_95647_MOESM1_ESM.zip › Supplementary/QQplot_1GSR_boredom - GSR_frustration.png]

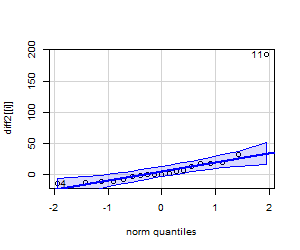

Supplement: Supplementary file 1 — Supplementary Material 1 [file 41598_2025_95647_MOESM1_ESM.zip › Supplementary/QQplot_1GSR_flow - GSR_frustration.png]

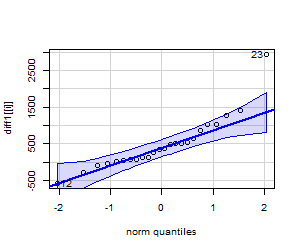

Supplement: Supplementary file 1 — Supplementary Material 1 [file 41598_2025_95647_MOESM1_ESM.zip › Supplementary/QQplot_1Gyro_std_boredom - Gyro_std_flow.png]

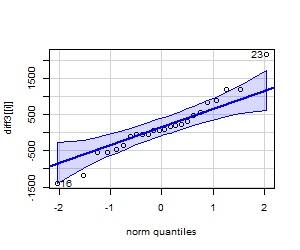

Supplement: Supplementary file 1 — Supplementary Material 1 [file 41598_2025_95647_MOESM1_ESM.zip › Supplementary/QQplot_1Gyro_std_boredom - Gyro_std_frustration.png]

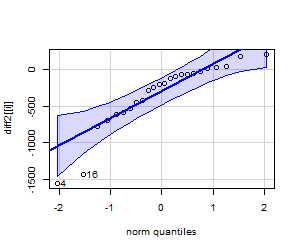

Supplement: Supplementary file 1 — Supplementary Material 1 [file 41598_2025_95647_MOESM1_ESM.zip › Supplementary/QQplot_1Gyro_std_flow - Gyro_std_frustration.png]

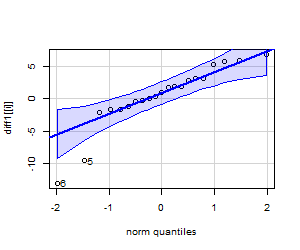

Supplement: Supplementary file 1 — Supplementary Material 1 [file 41598_2025_95647_MOESM1_ESM.zip › Supplementary/QQplot_1HR_boredom - HR_flow.png]

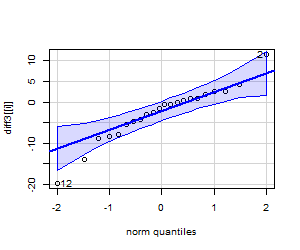

Supplement: Supplementary file 1 — Supplementary Material 1 [file 41598_2025_95647_MOESM1_ESM.zip › Supplementary/QQplot_1HR_boredom - HR_frustration.png]

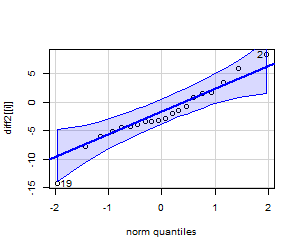

Supplement: Supplementary file 1 — Supplementary Material 1 [file 41598_2025_95647_MOESM1_ESM.zip › Supplementary/QQplot_1HR_flow - HR_frustration.png]

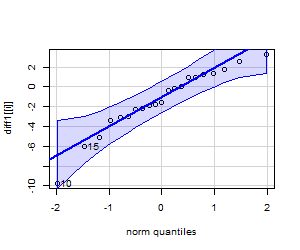

Supplement: Supplementary file 1 — Supplementary Material 1 [file 41598_2025_95647_MOESM1_ESM.zip › Supplementary/QQplot_1SPO2_boredom - SPO2_flow.png]

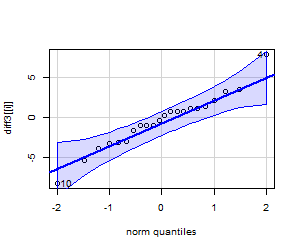

Supplement: Supplementary file 1 — Supplementary Material 1 [file 41598_2025_95647_MOESM1_ESM.zip › Supplementary/QQplot_1SPO2_boredom - SPO2_frustration.png]

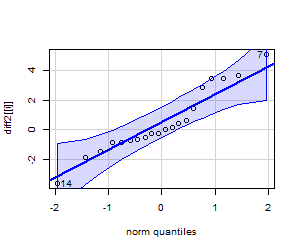

Supplement: Supplementary file 1 — Supplementary Material 1 [file 41598_2025_95647_MOESM1_ESM.zip › Supplementary/QQplot_1SPO2_flow - SPO2_frustration.png]

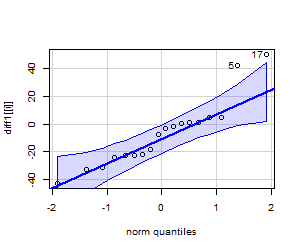

Supplement: Supplementary file 1 — Supplementary Material 1 [file 41598_2025_95647_MOESM1_ESM.zip › Supplementary/QQplot_20ch1_closed_beta_beta_boredom - ch1_closed_beta_beta_flow.png]

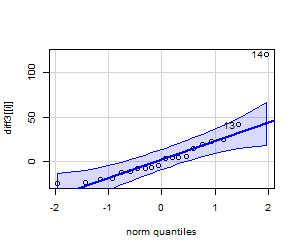

Supplement: Supplementary file 1 — Supplementary Material 1 [file 41598_2025_95647_MOESM1_ESM.zip › Supplementary/QQplot_20ch1_closed_beta_beta_boredom - ch1_closed_beta_beta_frustration.png]

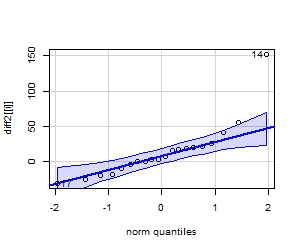

Supplement: Supplementary file 1 — Supplementary Material 1 [file 41598_2025_95647_MOESM1_ESM.zip › Supplementary/QQplot_20ch1_closed_beta_beta_flow - ch1_closed_beta_beta_frustration.png]

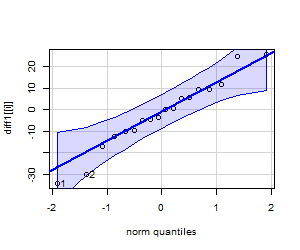

Supplement: Supplementary file 1 — Supplementary Material 1 [file 41598_2025_95647_MOESM1_ESM.zip › Supplementary/QQplot_21ch2_closed_delta_delta_boredom - ch2_closed_delta_delta_flow.png]

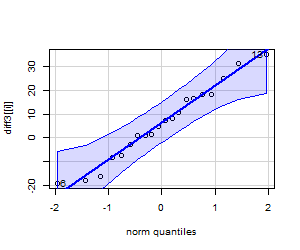

Supplement: Supplementary file 1 — Supplementary Material 1 [file 41598_2025_95647_MOESM1_ESM.zip › Supplementary/QQplot_21ch2_closed_delta_delta_boredom - ch2_closed_delta_delta_frustration.png]

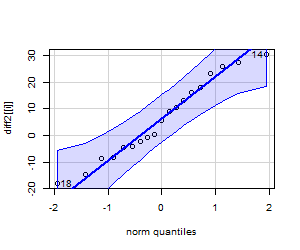

Supplement: Supplementary file 1 — Supplementary Material 1 [file 41598_2025_95647_MOESM1_ESM.zip › Supplementary/QQplot_21ch2_closed_delta_delta_flow - ch2_closed_delta_delta_frustration.png]

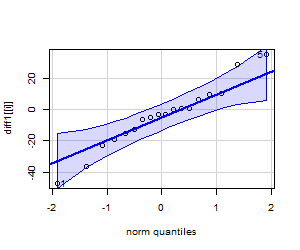

Supplement: Supplementary file 1 — Supplementary Material 1 [file 41598_2025_95647_MOESM1_ESM.zip › Supplementary/QQplot_22ch2_closed_theta_theta_boredom - ch2_closed_theta_theta_flow.png]

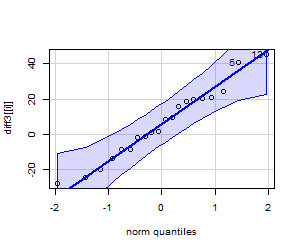

Supplement: Supplementary file 1 — Supplementary Material 1 [file 41598_2025_95647_MOESM1_ESM.zip › Supplementary/QQplot_22ch2_closed_theta_theta_boredom - ch2_closed_theta_theta_frustration.png]

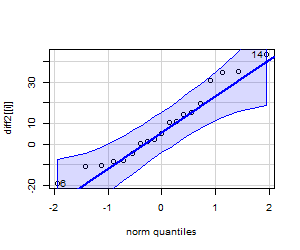

Supplement: Supplementary file 1 — Supplementary Material 1 [file 41598_2025_95647_MOESM1_ESM.zip › Supplementary/QQplot_22ch2_closed_theta_theta_flow - ch2_closed_theta_theta_frustration.png]

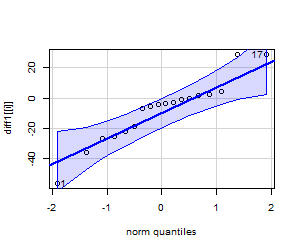

Supplement: Supplementary file 1 — Supplementary Material 1 [file 41598_2025_95647_MOESM1_ESM.zip › Supplementary/QQplot_23ch2_closed_alpha_alpha_boredom - ch2_closed_alpha_alpha_flow.png]

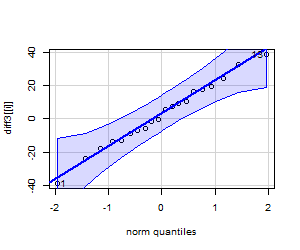

Supplement: Supplementary file 1 — Supplementary Material 1 [file 41598_2025_95647_MOESM1_ESM.zip › Supplementary/QQplot_23ch2_closed_alpha_alpha_boredom - ch2_closed_alpha_alpha_frustration.png]

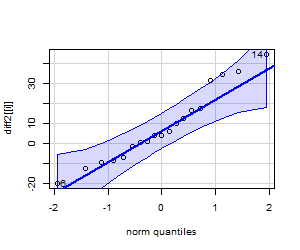

Supplement: Supplementary file 1 — Supplementary Material 1 [file 41598_2025_95647_MOESM1_ESM.zip › Supplementary/QQplot_23ch2_closed_alpha_alpha_flow - ch2_closed_alpha_alpha_frustration.png]

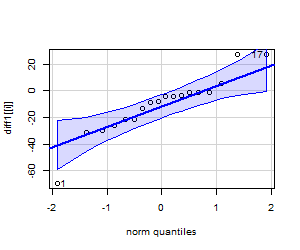

Supplement: Supplementary file 1 — Supplementary Material 1 [file 41598_2025_95647_MOESM1_ESM.zip › Supplementary/QQplot_24ch2_closed_beta_beta_boredom - ch2_closed_beta_beta_flow.png]

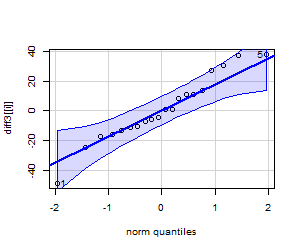

Supplement: Supplementary file 1 — Supplementary Material 1 [file 41598_2025_95647_MOESM1_ESM.zip › Supplementary/QQplot_24ch2_closed_beta_beta_boredom - ch2_closed_beta_beta_frustration.png]

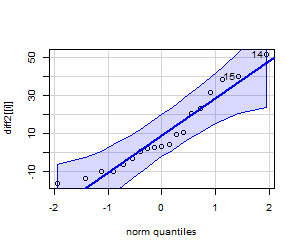

Supplement: Supplementary file 1 — Supplementary Material 1 [file 41598_2025_95647_MOESM1_ESM.zip › Supplementary/QQplot_24ch2_closed_beta_beta_flow - ch2_closed_beta_beta_frustration.png]

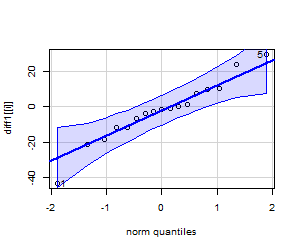

Supplement: Supplementary file 1 — Supplementary Material 1 [file 41598_2025_95647_MOESM1_ESM.zip › Supplementary/QQplot_25ch3_closed_delta_delta_boredom - ch3_closed_delta_delta_flow.png]

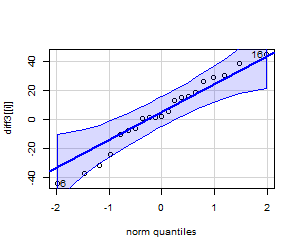

Supplement: Supplementary file 1 — Supplementary Material 1 [file 41598_2025_95647_MOESM1_ESM.zip › Supplementary/QQplot_25ch3_closed_delta_delta_boredom - ch3_closed_delta_delta_frustration.png]

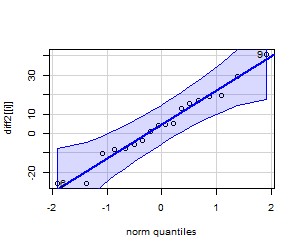

Supplement: Supplementary file 1 — Supplementary Material 1 [file 41598_2025_95647_MOESM1_ESM.zip › Supplementary/QQplot_25ch3_closed_delta_delta_flow - ch3_closed_delta_delta_frustration.png]

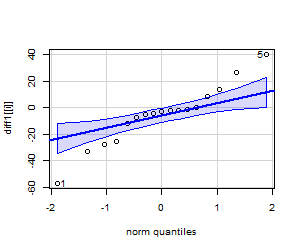

Supplement: Supplementary file 1 — Supplementary Material 1 [file 41598_2025_95647_MOESM1_ESM.zip › Supplementary/QQplot_26ch3_closed_theta_theta_boredom - ch3_closed_theta_theta_flow.png]

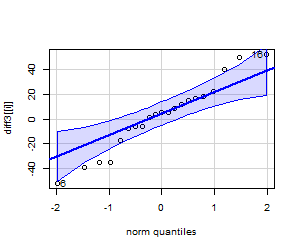

Supplement: Supplementary file 1 — Supplementary Material 1 [file 41598_2025_95647_MOESM1_ESM.zip › Supplementary/QQplot_26ch3_closed_theta_theta_boredom - ch3_closed_theta_theta_frustration.png]
